# CD1d facilitates African swine fever virus entry into the host cells via clathrin-mediated endocytosis

**DOI:** 10.1080/22221751.2023.2220575

**Published:** 2023-06-22

**Authors:** Xin Chen, Jun Zheng, Chuanxia Liu, Tingting Li, Xiao Wang, Xuewen Li, Miaofei Bao, Jiangnan Li, Li Huang, Zhaoxia Zhang, Zhigao Bu, Changjiang Weng

**Affiliations:** a Division of Fundamental Immunology, National African Swine Fever Para-reference Laboratory, State Key Laboratory for Animal Disease Control and Prevention, Harbin Veterinary Research Institute, Chinese Academy of Agricultural Sciences (CAAS), Harbin, People’s Republic of China; b Heilongjiang Provincial Key Laboratory of Veterinary Immunology, Harbin, People’s Republic of China

**Keywords:** African swine fever virus, CD1d, virus entry, clathrin-mediated endocytosis, EPS15

## Abstract

African swine fever (ASF) is a highly contagious and acute hemorrhagic viral disease with high morbidity and mortality in domestic pigs and wild boars. The disease has become a global threat to the pig production industry and has caused enormous economic losses in many countries in recent years. However, the molecular mechanism underlying ASF virus (ASFV) entry of the host cells is not fully understood, which restricts the development of vaccines and antiviral-drugs of ASFV. In this study, we found that the host protein CD1d acts as a host factor, which mediates ASFV entry into the host cells. As the main capsid protein on the surface of ASFV virions, p72 can mediate viral entry. Using IP-MS assay, CD1d was identified as a binding partner of p72 on surface of ASFV virions. Knockdown of CD1d expression and blocking the cells with anti-pCD1d antibody, or incubating ASFV virions with soluble CD1d protein could significantly inhibit ASFV infection. CD1d is located on the membrane surface of primary porcine alveolar macrophages (PAMs) and mediates the virus entry via binding to p72. CD1d knockout or CD1d knockdown assay showed that CD1d could facilitate ASFV virions internalization via clathrin-mediated endocytosis (CME). Furthermore, CD1d interacts with EPS15 to mediate ASFV entry via clathrin-mediated endocytosis. Overall, our findings revealed that CD1d is a novel host-entry factor involved in ASFV internalization via the EPS15-clathrin endocytosis axis and a potential target for antiviral intervention.

## Introduction

ASFV, the only member of the *Asfarviridae* family, genus *Asfivirus*, is a large and double-stranded DNA arbovirus. ASFV infects different species and ages of swine and provokes severe economic and expansion threats [1,2]. ASFV genomes vary in length between 170 and 190 kbp, containing between 151 and 167 open reading frames (ORFs). About half of ASFV proteins lack any known or predicted function [3,4]. More than 150 viral proteins are expressed in ASFV-infected macrophages or other cell lines, which not only function to facilitate virus entry, replication, virion assembly, and egress [5], but also regulate host antiviral innate immune responses [6]. Due to the large structure of ASFV virions and the complex mechanism involved in the evasion of innate immune responses, there are no treatment drugs or effective vaccines commercially available. Until now, the prevention and control of African swine fever (ASF) still depend on the implementation of rigorous import policies and biosecurity measures [7].

The ASFV virions are icosahedral morphology composed of four concentric domains, including nucleoid, core–shell, inner lipid envelope and capsid from the inner to the outer layers. The mature virions acquire an additional external lipid envelope when viruses bud out through the plasma membrane [8]. Previous studies showed that the external lipid envelopes of ASFV virions are not necessary for ASFV infectivity [4,9,10], suggesting that the entry of ASFV into the target cell may depend on the role of viral capsid proteins on the surface of ASFV virions. It has been reported that the capsid proteins control the initial phases of viral infection, including virus attachment, endocytosis, and genome release into the host cells. The known capsid proteins of ASFV virions mainly contain p72 (pB646L), p49 (pB438L) and pE120R. Among them, p72 is the most dominant structural component of ASFV virions and it accounts for 31–33% of the total mass [3,11]. As one of the major antigens of ASFV, p72 is relatively conserved, and several monoclonal antibodies against p72 were found to have neutralizing ability, which can significantly affect the infection of ASFV [12–15].

Viral infection is a very complex process that begins with the attachment of virions by binding host factors on the cell membrane, followed by virions’ internalization into targeting cells in a variety of pathways. Clathrin-mediated endocytosis (CME) is a common pathway used by many viruses to mediate viral entry [16–20]. In addition to the involvement of clathrin, this process requires various adaptor proteins, including clathrin-associated adaptor complex-2 (AP-2), EPS15, Epsin1, and AP180, which contribute to cargo selection, coat assembly, and maturation of clathrin-coated buds [21]. AP-2 is a multimeric protein that functions as a core that connects the cargo, cell membrane components, clathrin, and other proteins involved in clathrin-coated vesicle formation [22]. The α and β subunits of AP-2 provide binding sites for various clathrin adaptors (such as EPS15, Epsin1, and AP180) to form a complex, leading to nucleation by clathrin-coated pits [21]. After entering the targeting cells, the virions in the endocytosis vesicles can be transported via the early endosomes (EE) and late endosomes (LE) to the lysosomes (LY) for capsid protein degradation, resulting in the release of the viral genome to the cytoplasm for viral replication [23–25]. Recently, researchers reported that the interaction between the internal envelope proteins E248R and E199L of ASFV and the Nieman-Pick C protein of host endosomal proteins is required for the virus endosomal membrane to fuse with the endosomal membrane, resulting in releasing of virus core particles into the cytoplasm to initiate infection [26].

In the present study, we established an elaborate labelling method showing that p72 on the surface of ASFV virions is attached on the cell membrane and involved in ASFV entry. Subsequently, we found that the host protein CD1d is a new binding partner of ASFV p72. Knockdown of CD1d expression in PAMs or knockout of CD1d gene in MA104 cells greatly reduced ASFV infection while overexpression of CD1d significantly increased ASFV infection. Furthermore, we found that CD1d interacted with an adaptor protein EPS15, which promotes the formation of EPS15-AP-2-clathrin complexes, resulting in ASFV endocytosis in PAMs. Collectively, our findings shed light on the molecular events involved in ASFV entry into the permissive cells.

## Materials and methods

### Ethics statements

All experiments with ASFV isolate Pig/HLJ/2018 (ASFV HLJ/2018) and rASFV-Gluc-GFP were conducted within the enhanced biosafety level 3 (P3) facilities at the Harbin Veterinary Research Institute (HVRI) of the Chinese Academy of Agricultural Sciences (CAAS) and were approved by the Ministry of Agriculture and Rural Affairs and China National Accreditation Service for Conformity Assessment.

### Cell and viruses

PAMs were prepared from lung lavage of 4-week-old specific-pathogen-free (SPF) piglets, as previously described [27] and maintained in Roswell Park Memorial Institute 1640 medium (RPMI 1640) supplemented with 10% heat-inactivated fetal bovine serum (FBS; Gibco, Carlsbad, CA), 100 U/mL penicillin, 100 mg/mL streptomycin. HEK293 T cells and MA104 cells obtained from the American Type Culture Collection (ATCC) were cultured in Dulbecco’s modified Eagle’s medium (DMEM) supplemented with 10% FBS and penicillin–streptomycin. All the cells were maintained at 37°C with 5% CO_2_. The ASFV isolates HLJ/2018 (GenBank accession number: MK333180.1) were isolated from the pigs, as previously described [28]. A recombinant ASFV expressing Gaussia luciferase (Gluc) and GFP (rASFV-Gluc-GFP) was generated from ASFV HLJ/18 isolate and maintained in our laboratory [29]. In brief, two reporter expression boxes expressing Gluc and GFP were inserted into the *K145R* gene site of ASFV using CRISPR/Cas9 gene editing and homologous recombination techniques. There was no difference in growth kinetics between the parent virus and the recombinant virus. HSV-1 was kindly provided by Prof. Hongbing Shu (Wuhan University, China). VSV was kindly provided by Prof. Bo Zhong (Wuhan University, China). The PRV-TJ and PRRSV HuN4 strains are maintained in our institute (HVRI, China).

### Antibodies, proteins and reagents

Mouse anti-HA monoclonal antibodies and rabbit anti-HA polyclonal antibodies were purchased from Cell Signaling Technology (America). Rabbit anti-GAPDH, rabbit anti-Clathrin heavy chain (CHC), rabbit anti-EPS15, rabbit anti-AP2A2, rabbit anti-AP2B1, rabbit anti-AP2M1, rabbit anti-AP180, rabbit anti-CD1a, and rabbit anti-CD1d antibodies were purchased from Proteintech (Wuhan, China). Rabbit anti-Epsin1 and rabbit anti-CD1b antibodies were purchased from Abcam (England). Mouse anti-p72 antibody was purchased from Zoonogen (Beijing, China). The cDNA of porcine CD1d protein (GenBank accession number: XP_005663291.1) was synthesized by GenScript and cloned into expression vector pET-21a (+). After the pET-21a-CD1d plasmid was transformed into *E. coli* BL21 (DE3), recombinant porcine CD1d protein was expressed and purified as previously described [27]. Rabbit anti-pCD1d antibody was prepared by immunizing the rabbits with recombinant porcine CD1d protein as previously described [27]. Mouse anti-p34 antibody and mouse anti-pB318L antibody were generated as previously described [27] and maintained in our laboratory. IRDye 800CW goat anti-mouse IgG (H + L) was purchased from Sera Care, and IRDye 800CW goat anti-rabbit IgG (H + L) was purchased from LI-COR. Mem-PER™ Plus Membrane protein extraction kit was purchased from Thermo Fisher. Chlorpromazine hydrochloride (CPZ) was purchased from Sigma-Aldrich.

### Co-immunoprecipitation (Co-IP) and Western blot

Co-IP and Western blot analysis was performed, as described previously [30]. The cells were collected and lysed in 1% NP-40 cell lysis buffer with fresh protease inhibitors. Then the lysates were immunoprecipitated by anti-Flag (M2) agarose beads (Sigma) overnight at 4°C on a roller. To test the interaction of endogenous proteins, PAMs were mock infected or infected with ASFV-HLJ/18 (MOI = 5) for 24 h. The cell lysates then were incubated with anti-p72 antibody or IgG for 8 h at 4°C, and the proteins were captured using protein A + G Sepharose (Thermo). The cell lysates and immunoprecipitants were resolved by 12% SDS-PAGE and then transferred to polyvinylidene difluoride membranes (ISEQ00010, Merck Millipore) for immunoblot analysis.

### Mass spectrometry

The ASFV p72-binding proteins in the immunoprecipitants were resolved by 12% SDS-PAGE and silver staining (Solarbio, China) gel was cut and processed for liquid chromatography-tandem mass spectrometry (LC-MS/MS) (Probability-based protein identification by searching sequence databases using mass spectrometry data). The MS/MS signals were then processed against the National Center for Biotechnology Information (NCBI) protein database by using the Mascot Server (Matrix Science). High-confidence peptides with a prerequisite of a minimum of two peptides leading to the identification of proteins were selected and listed in Supplementary Table 1. The protein “score” displays the standard score, the cumulative protein score based on summing the ion scores of the unique peptides. A higher score indicates higher confidence in identification.

### Immunofluorescence assays (IFA)

Cells grown on a poly-L-Lysine-coated glass-bottom cell culture dish were fixed in 4% paraformaldehyde for 30 min and washed one time with 1×PBS (pH = 7.4). The Cells were then permeabilized in 0.2% Triton X-100 in 1 × PBS and blocked with 5% BSA in 1×PBS for 1 h. Then cells were incubated with anti-Flag and anti-HA antibodies for 1 h and then stained with indicated secondary antibodies for 1 h. Nuclear DNA staining was performed with DAPI (Sigma). Samples were visualized with a Zeiss LSM-800 laser scanning fluorescence microscope (Carl Zeiss AG, Oberkochen, Germany). For the ASFV attachment assay, non-permeabilized cells were used and detected with IFA. Then cells were incubated with mouse anti-p72 antibody. For assessment of the colocalization of p72 and CD1d, PAMs were infected for 24 h, and the cells were incubated with mouse anti-p72 antibody and rabbit anti-CD1d antibody. Colocalization analyses were performed using the Pearson correlation coefficient showing an actual overlap of the signals, which is considered to represent the true degree of two proteins’ colocalization.

### ASFV infection and RNA interference

PAMs were seeded into 24-well plates at 2 × 10^5^ cells per well and infected with ASFV (MOI = 1) for 1, 2, 4, 8, 24, and 48 h, respectively. At different time points, the cells were harvested. All siRNAs and siRNA controls (si-Ctrl) were designed and synthesized by Sangon (Shanghai, China) and listed in Supplementary Table 2. In knockdown experiments, PAMs were transfected with siRNA negative controls or siRNAs as indicated at a final concentration of 10 nM using Lipofectamine RNAiMAX according to the manufacturer’s instructions. Twenty-four hours after transfection, cells were infected with ASFV (MOI = 1) for 24 h. The total RNA and DNA of cells were extracted for quantitative polymerase chain reaction (qPCR).

### Quantitative polymerase chain reaction (qPCR)

PAMs were collected, and total RNAs were extracted using TRIzol reagent (Invitrogen, Carlsbad, CA, USA). The reverse transcription cDNAs were prepared by using PrimeScript™ RT Reagent Kit (Takara, Kusatsu, Shiga, Japan) according to the manufacturer’s instructions. To detect CD1d, CHC, EPS15, CD1a, CD1b, AP2A2, AP2B1, AP2M1, AP180, and Epsin1 mRNA expression, the cDNAs were amplified using a QuantStudio 5 system (Applied Biosystems, Sunnyvale, CA, USA) with SYBR® Premix ExTaq™ II (Takara, Kusatsu, Shiga, Japan). The relative mRNA levels of these genes were evaluated by the 2^−ΔΔCT^ method using Hypoxanthine phosphoribosyl transferase (HPRT) mRNA as an endogenous control. The primers for RT-qPCR are listed in Supplementary Table 2. For ASFV genomic DNA copy detection, ASFV genomic DNA was extracted from cells using a Qiagen DNA Mini Kit (Qiagen, Germany). The qPCR was carried out on a QuantStudio 5 system according to the OIE-recommended procedure [31].

### Luciferase reporter gene assay

Gaussian luciferase activities were measured with a Luciferase Reporter Assay System (Pierce^TM^ Gaussia Luciferase Flash Assay Kit) according to the manufacturer’s instructions.

### Antibody blocking and virus neutralization assay

PAMs were seeded in 48-well plates overnight and then incubated with different concentrations of anti-pCD1d antibody for 1 h at 4°C. After being washed with fresh media, the cells were infected with rASFV-Gluc-GFP (MOI = 1) for 24 h. As a control, PAMs were pretreated in parallel with rabbit IgG (Proteintech). The cells were harvested for total DNA extraction and ASFV genomic DNA copy detection or analysed by flow cytometry analysis. ASFV was incubated with different concentrations of CD1d protein (Proteintech) or anti-p72 antibody for 4 h at 4°C. Then, PAMs were infected with virus-CD1d or virus-antibody mixtures for 24 h. ASFV was preincubated with BSA or mouse IgG (Proteintech) was used as a control. Then, the cells were harvested and measured as previously described.

### Generation CD1d knockout cell line by CRISPR/Cas9

CD1d knockout cell lines were generated using CRISPR/Cas9 technology. A guide RNA (gRNA) with high target specificity was selected using the CRISPR design tool from E-CRISP (https://www.e-crisp.org/E-CRISP). The gRNA (GGACGAGGGAAACAGTGCAG) was cloned in the pMD-EGFP-Cas9-U6 plasmid. MA104 cells were transfected with a pMD-EGFP-Cas9-U6-gRNA plasmid to generate CD1d knockout clones by single-cell sorting of EGFP-positive MA104 cells. After about three weeks, MA104-ΔCD1d cell lines were selected by genome sequencing analysis and then analysed by flow cytometry.

### Establishment of stable cell lines overexpressing CD1d

The cDNA of the porcine CD1d sequence (NCBI Reference Sequence: XM_005663234.3) was cloned into a pLVX-IRES-Puro vector to generate lentivirus via the psPAX2-pMD2G system in HEK293 T cells. MA104-ΔCD1d cells were transduced with packaged the lentivirus expressing CD1d and cultured in a medium supplemented with puromycin (Gibco) for selection. The surviving cell clone was isolated, propagated, and examined for stable expression of CD1d by western blot.

### Virus binding and internalization assay

MA104 cells were infected with ASFV (MOI = 2) and incubated at 4°C for 1 h. The unbound virus was removed by twice washes with 1× PBS. The dishes were overlaid with a complete medium and shifted to 37°C for 2 h. The infected cells were treated with 0.2 M Glycine (pH = 3) for 2 min at room temperature to inactivate extracellular viruses and then washed twice with 1× PBS. Cells were harvested for total DNA extraction and ASFV genomic copies detection. As a control for each experiment, adsorbed viruses were removed using acid glycine washes, and shown in the experiments as 4°C + Gly.

### Preparation of DiD-labelled ASFV (DiD-ASFV) for binding and internalization

To prepare DiD-ASFV, the purified ASFV HLJ/2018 particles were incubated with 100 nM DiD (Thermo) in 1× PBS for 1 h at room temperature and then centrifuged at 5000 × g for 1 h at 4°C to remove the supernatant through Amicon Ultra (UFC9100, Merck Millipore). The sediment was washed with 1× PBS twice and resuspended in 1×PBS and named DiD-ASFV. The PAMs were infected with DiD-ASFV at 4°C for 1 h, washed to remove the free DiD-ASFV, and then transferred to 37°C to allow incorporation of DiD-ASFV up to 1 h. The cells were incubated with anti-p72 antibody or not and observed using high-magnification confocal microscopy for DiD-ASFV binding and internalization. To quantify the binding and internalization of DiD-ASFV, we used WGA-labelled cell membranes (Grey) as a reference, where colocalized (Red) with the cell membrane was the binding virus, and internal (Red) was the internalized virus. More than two hundred cells were counted per sample, and the percentage of cells that internalized DiD-ASFV positive was calculated.

### Staining of CD1d and analysed by flow cytometry analysis

PAMs or MA104 cells were collected with trypsin digestion and washed twice with 1× PBS. Then the respective cells were blocked with 5% BSA in 1× PBS and incubated with rabbit anti-pCD1d antibody or rabbit anti-CD1d antibody (1 μg/mL), followed by a secondary goat anti-rabbit antibody conjugated to Alexa Fluor 488. The cells were subjected to flow cytometry analysis. Ten thousand events were registered for each experiment and cellular fluorescence was quantitated as the mean fluorescence intensity.

### Chlorpromazine (CPZ) inhibitor assay

PAMs or MA104 cells were treated with different concentrations (5, 10, and 20 μM) of CPZ for 30 min, then infected with ASFV HLJ/2018 (MOI = 1) for 1 h at 4°C. Next, the cells were washed to remove any unbound ASFV and incubated at 37°C for 2 h. DMSO was used as a control. The total DNA of cells was extracted for the detection of ASFV genomic DNA copies.

### Transferrin and Dextran uptake assays

PAMs or MA104 cells were serum starved for 30 min before incubation with 50 μg/mL Cy3-labelled transferrin (Molecular Probes) in DMEM for 20 min at 4°C for binding. The cells were then washed with cold 1× PBS and transferred to 37°C for 15 min. Residual and non-internalized labelled transferrin was removed by acid washing with 0.2 M Glycine (pH = 3). Dextran uptake assay was performed using Alexa Fluor-594-labelled dextran (Molecular Probes). The starved cells were incubated with 0.5 mg/mL Alexa Fluor-594-labelled-dextran for 10 min at 37°C. The cells were then washed three times with cold 1× PBS and residual and non-internalized Alexa Fluor-594-labelled dextran was removed by 0.1 M sodium acetate (pH = 5.5). The cells were then processed for immunoﬂuorescence and examined by confocal microscopy.

### Statistical analysis

In the present study, GraphPad Prism 8.0 was used for statistical calculations and data plotting. Differences between two independent samples were evaluated by two-tailed Student’s *t*-tests. Differences between multiple samples were analysed by one-way analysis of variance (ANOVA). The data are presented as the mean ± SD. We considered *P *< 0.05 to be statistically significant. Significance values were set as follows: ns (not significant), *P *> 0.05; ****, *P *< 0.0001; ***, *P *< 0.001; **, *P *< 0.01; *, *P *< 0.05.

## Results

### ASFV p72 is involved in ASFV entry and interacts with a novel partner CD1d

**Figure 1.**
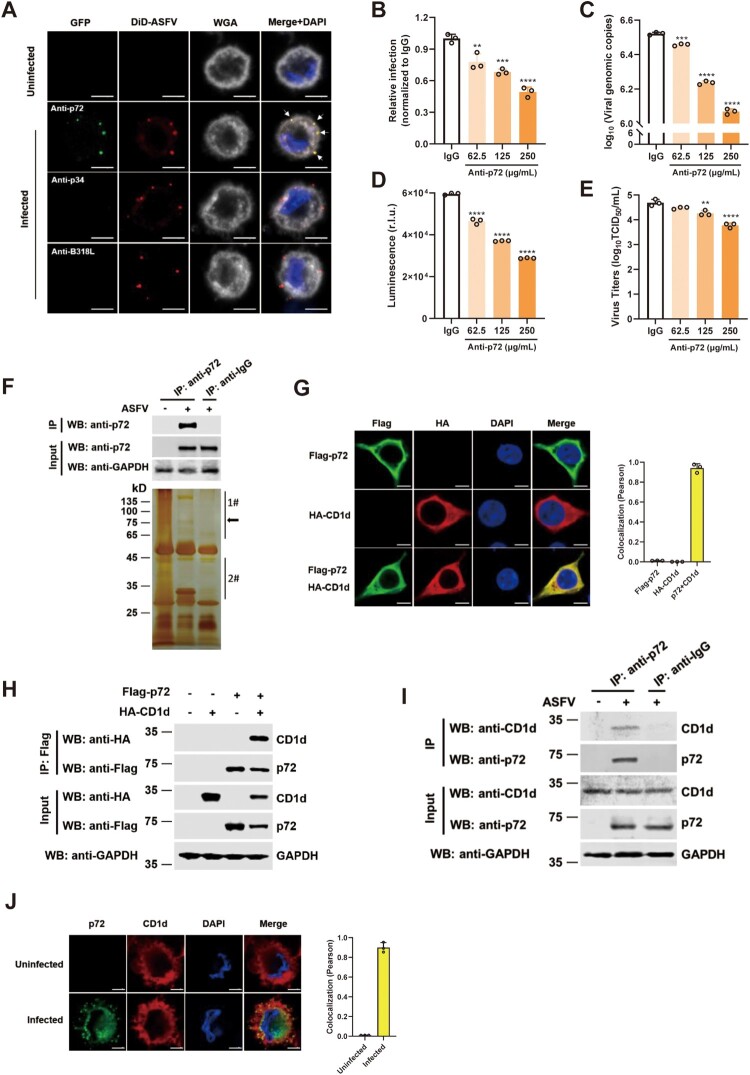
ASFV p72 facilitates viral entry and interacts with CD1d protein. (A) Immunocytochemistry was performed to detect the co-localization of DiD-ASFV (Red) and p72 (Green) after virus infection of PAMs for 1 h at 4°C. As negative controls, co-localization of DiD-ASFV and p34 or pB318L were also detected. The cell membrane was stained with WGA (Grey). The nuclei were stained with DAPI (Blue). White arrows show the co-localized DiD-ASFV and p72. Bar = 5 μm. (B–E) ASFV virions were incubated with anti-p72, anti-p34 and anti-pB318L antibodies at the indicated concentrations for 4 h at 4°C. Then, PAMs were infected with 1 MOI rASFV-Gluc-GFP virions-antibody mixtures for 24 h. The infection rate of ASFV was determined by flow cytometry analysis (B), the genomic DNA level of the virus was determined by qPCR (C) and luciferase activity (D), and the virus titre in the culture supernatant was determined by TCID_50_ assay (E). The data were analysed statistically using one-way analysis of variance (ANOVA). The results are presented as the mean ± standard deviation of three independent measurements. (F) Co-IP and silver staining were used to detect the expression of endogenous p72 in PAMs and the enriched p72-binding proteins. PAMs were mock infected or infected with ASFV-HLJ/18 (MOI = 5) for 24 h. The cell lysates then were incubated with anti-p72 antibody or mouse IgG for 8 h at 4°C. ASFV p72-binding proteins were eluted from protein A + G Sepharose and analysed on SDS-PAGE followed by silver staining. The p72 band is indicated by a black arrow. The 1# and 2# indicate that the specific bands containing regions were cut for mass spectrometry analysis. (G) HEK293T cells were co-transfected with plasmids expressing Flag-p72 and HA-CD1d. p72 (Green) and CD1d (Red) were detected by immunofluorescence staining with anti-Flag or anti-HA antibodies, respectively. Nuclei were stained by DAPI. Co-localization of p72 and CD1d was performed using the Pearson correlation coefficient. Bar = 5 μm. (H) The cell lysates from HEK293T cells co-transfected with Flag-p72 and HA-CD1d were subjected to co-immunoprecipitation with an anti-Flag antibody. Immuno-precipitates and whole-cell lysates (Input) were immunoblotted with anti-Flag and anti-HA antibodies, respectively. (I) PAMs were mock infected or infected with ASFV HLJ/2018 at an MOI of 5, and the interaction of endogenous CD1d and p72 in the cell lysates was detected by Co-IP with antibodies against p72, CD1d or IgG and protein A + G Sepharose. (J) PAMs were mock infected or infected with ASFV HLJ/2018 at an MOI of 5, and the localization of endogenous CD1d and p72 was detected by immunofluorescence staining with anti-p72 and anti-CD1d antibodies, respectively. Nuclei were stained by DAPI. Colocalization of p72 and CD1d was performed using the Pearson correlation coefficient. Bar = 5 μm. ****P < 0.0001; ***P < 0.001; **P < 0.01.

As the main capsid protein on the surface of ASFV virions, p72 is one of the first identified viral proteins that are responsible for the induction of neutralizing antibodies to affect ASFV attachment to macrophages [32,33]. And it was reported in 1996 that antibodies against p72 are involved in the inhibition of a first step of the replication cycle related to virus attachment [14]. Thus, we proposed that p72 may interact with viral entry-related receptor(s) on the cell membrane to mediate viral entry. Therefore, we first tested whether ASFV p72 can be used as an exploratory molecule to identify the ASFV receptor(s). Mature ASFV virions were purified from the culture supernatant of ASFV HLJ/2018 (ASFV-WT) by sucrose density gradient centrifugation and then labelled with DiD (1,1-Dioctadecyl-3, 3, 3, 3-tetramethylindodicarbocyanine, 4-Chlorobenzenesulfonate Salt, a lipophilic carbocyanine dye). PAMs were infected with DiD-ASFV and examined by immunofluorescence microscopy. We found that DiD-ASFV adhered to the cell membrane when PAMs were incubated with DiD-ASFV virions for 1 h at 4°C. In addition, we noticed that p72 also colocalized with ASFV virions on the cell membrane (Figure 1(A)). ASFV p34 is a structural protein cleaved from pp220 polyprotein by pS273R and assembled into the core shell of ASFV [34]. ASFV pB318L, a pentenyl transferase encoded by the *B318L* gene of ASFV, is mainly presented at the cytoplasmic virus assembly sites and is associated with precursor viral membranes from the endoplasmic reticulum [35]. As negative controls, anti-p34 and anti-pB318L antibodies could not label ASFV virions on the cell membrane because they are not on the surface of ASFV virions. To test whether anti-p72, anti-p34, and anti-pB318L antibodies can inhibit ASFV entry, rASFV-Gluc-GFP virions were incubated with these antibodies with indicated concentration for 4 h at 4°C, respectively. Then, PAMs were infected with the mixtures for 24 h [29]. We found that different concentrations of anti-p72 antibodies significantly reduced ASFV infection in a dose-dependent manner (Figure 1(B–E)). However, anti-p34 and anti-pB318L antibodies did not inhibit ASFV infection (Supplementary Figure 1(A–I)). Taken together, these results show that p72 may mediate ASFV entry via interaction with host factor(s) on the cell membrane.

To identify p72-interacting proteins, PAMs were infected with ASFV-WT and then an immunoprecipitation assay was performed using an anti-p72 antibody. Silver staining results showed that several specific bands were observed in the anti-p72 antibody-precipitated lane in comparison with the anti-IgG control lane (Figure 1(F)). By analysing the results of mass spectrometry (MS), many potential p72-interacting proteins were identified (Supplementary Table 1). The “scores” of these identified proteins are the sum of the scores of the individual peptides identified, and a higher score indicates higher confidence in the identification. Among them, CD1d was identified as a p72 binding partner with the highest score. Therefore, in this study, we focus on CD1d for further investigation. CD1d is an MHC-class-I-like glycoprotein, which can present both foreign and self-lipids as cognate antigens to T cells [36]. The amino acid sequence of CD1d is shown in Supplementary Figure 1(J), together with four matched peptides detected in the MS analysis (highlighted in red).

To further confirm the interaction between CD1d and p72, indirect fluorescent assay (IFA) and co-immunoprecipitation (Co-IP) were performed. The results showed that CD1d colocalized and immunoprecipitated with p72 (Figure 1(G,H) and Supplementary Figure 1(K)). Next, we tested whether endogenous CD1d interacts with p72 during ASFV infection, PAMs were infected with ASFV-WT for 24 h. As a result, endogenous CD1d also co-immunoprecipitated and colocalized with ASFV p72 in PAMs (Figure 1(I, J)). These results indicate that ASFV p72 interacts with a novel partner CD1d.

### CD1d plays an important role for ASFV entry via binding to p72

**Figure 2.**
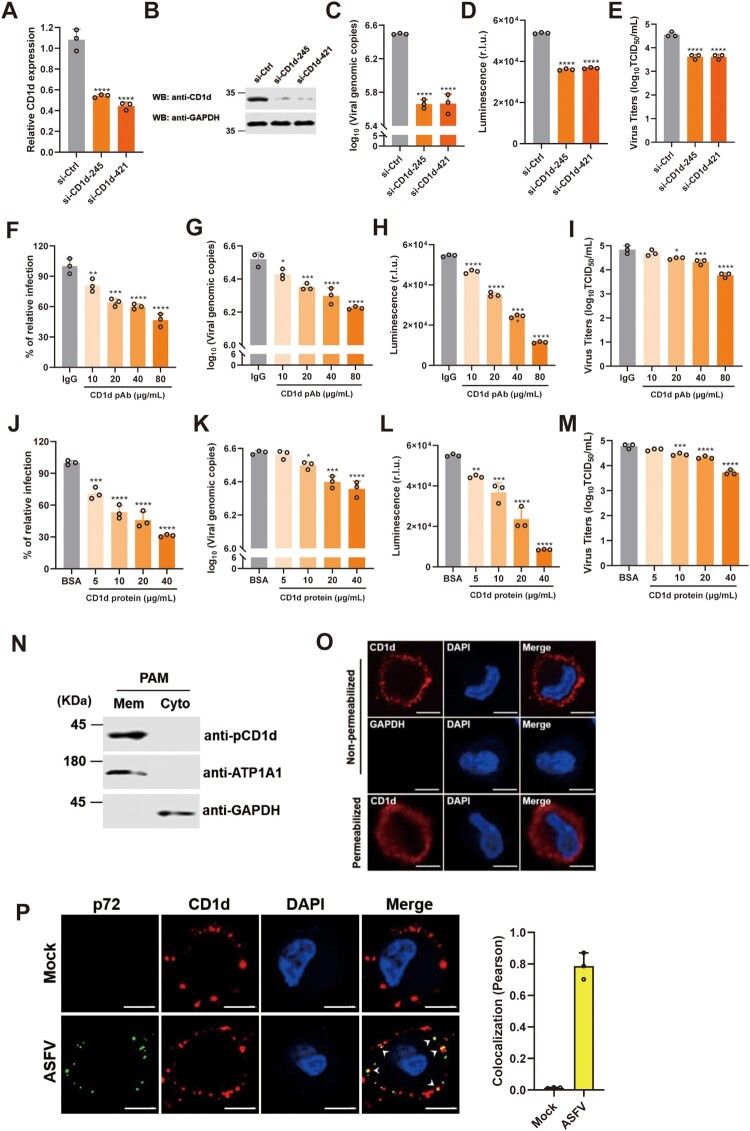
CD1d plays an important role for ASFV entry via binding to p72. (A–E) PAMs were transfected with scrambled si-Ctrl, and two specific RNAs targeting CD1d (si-CD1d-245 and si-CD1d-421) for 24 h, respectively. Then, the cells were infected with 1 MOI of rASFV-Gluc-GFP at 37°C for 24 h, the mRNA level of CD1d was examined by qPCR (A) and the protein level of CD1d was examined by Western blot (B). The genomic DNA level of ASFV in cells was determined by qPCR (C) and luciferase activity (D), and the virus titre in the culture supernatant was determined by TCID_50_ assay (E). (F–I) PAMs were incubated with anti-pCD1d antibody first at the indicated concentrations or IgG as a negative control for 1 h at 4°C, followed by infection with rASFV-Gluc-GFP at an MOI of 1. At 24 hpi, the infection rate of the ASFV was determined by flow cytometry analysis (F), the genomic DNA level of the ASFV in cells was determined by qPCR (G) and luciferase activity (H), and the infectious-virus titre in the culture supernatant was determined by TCID_50_ assay (I). (J–M) 1 MOI of rASFV-Gluc-GFP virions was preincubated with recombinant CD1d protein at the indicated concentrations or BSA as a negative control for 4 h at 4°C. Then, the rASFV-Gluc-GFP virions and protein mixtures were used to infect PAMs for 24 h. The infection level of the ASFV was determined by flow cytometry analysis (J), the genome level of the ASFV in cells was determined by qPCR (K) and luciferase activity (L), and the virus titre in the culture supernatant was determined by TCID_50_ assay (M). The data were analysed statistically using one-way analysis of variance (ANOVA). The results are presented as the mean ± standard deviation of three independent measurements. ****P < 0.0001; ***P < 0.001; **P < 0.01; *P < 0.05. (N) PAMs were isolated from the cell membrane and cytoplasm according to the instructions (Thermo), and CD1d expression was detected using the anti-CD1d antibodies. Meanwhile, ATP1A1 was detected as a maker of cell membranes and GAPDH was detected as a maker of cytoplasm. (O) PAMs were fixed with 4% paraformaldehyde with or without Triton X-100 and immunostained with anti-CD1d antibodies or anti-GAPDH antibodies, respectively. The nuclei were stained with DAPI (Blue). Bar = 5 μm. (P) Immunocytochemistry was performed to detect the co-localization of p72 (Green) and CD1d (Red) in PAMs that mock-infection or infected with ASFV infection for 1 h at 4°C. The nuclei were stained with DAPI (Blue). White arrows show the co-localized p72 and CD1d. Colocalization of p72 and CD1d was performed using the Pearson correlation coefficient. Bar = 5 μm.

To determine the effect of endogenous CD1d expression on ASFV infection, knockdown of CD1d expression in PAMs was performed by transfecting with specific siRNAs. We found that knockdown of CD1d expression in PAMs impaired viral genomic DNA production in cells and virus titre in the culture supernatant (Figure 2(A–E)). To further confirm the effect of CD1d during ASFV infection, we first incubated PAMs with anti-pCD1d antibodies and then infected with rASFV-Gluc-GFP. The results showed that the levels of viral infection, viral genomic DNA production in cells and virus titre in the culture supernatant were significantly decreased in a dose-dependent manner (Figure 2(F–I)), suggesting that ASFV infection could be blocked by the anti-pCD1d antibodies. In reverse, we incubated the rASFV-Gluc-GFP virions first with purified recombinant CD1d protein and then the mixtures were used to infect the PAMs. Similarly, we found that the pre-incubation with CD1d significantly reduced the ASFV infectability in PAMs in a dose-dependent manner (Figure 2(J–M)). Taken together, these results suggest that CD1d plays an important role in PAMs infection with ASFV, especially in the early stages of virus entry.

To investigate whether CD1d exists on the surface of PAMs, we isolated the membrane and cytoplasm of PAMs and detected CD1d expression, respectively. The results showed that like cell membrane protein ATP1A1 [37], CD1d was also mainly expressed on the cell membrane (Figure 2(N)). In addition, IFA was carried out using anti-pCD1d antibodies under permeabilized or non-permeabilized conditions. As shown in Figure 2(O), we found that CD1d protein was observed on the surface of PAMs without permeabilization, while the GAPDH in the cytoplasmic could not be detected by anti-GAPDH antibodies. In contrast, in permeabilized cells treated with 0.2% Triton X-100, CD1d proteins were found not only on the cell surface but also in the cytoplasm (Figure 2(O)). Further, we verified the subcellular localization of CD1d and ASFV p72 in the early step of ASFV entry (Figure 2(P)). The results showed that CD1d was colocalized with ASFV p72 when the viruses adhered to PAMs at 4°C for 1 h under non-permeabilized conditions, indicating that CD1d plays important roles in ASFV entry via binding to p72.

### CD1d is involved in the internalization of ASFV virions

**Figure 3.**
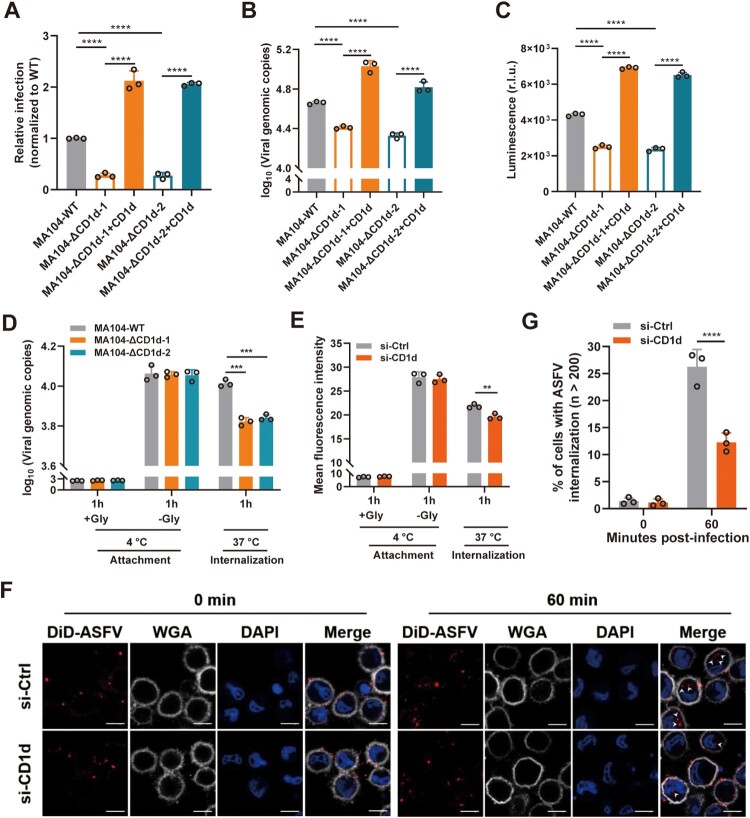
CD1d is involved in ASFV internalization. (A-C) MA104-WT and MA104-ΔCD1d cells were infected with rASFV-Gluc-GFP at an MOI of 1. At 24 hpi, the infection level of ASFV was determined by flow cytometry analysis (A), the genome level of the virus in cells was determined by qPCR (B) and luciferase activity (C). (D) MA104 and MA104-ΔCD1d cells were infected at 4°C with ASFV HLJ/2018 at an MOI of 2. After 1 h, cells were washed and collected for viral genome level (qPCR). Additional samples were then incubated at 37°C for 1 h, cells were washed and collected for viral genome level (qPCR). Incubation at 4°C with glycine (+Gly) represents the removement of the unbound virus from the cell surface. (E) PAMs were transfected with scrambled siRNA (si-Ctrl) and si-CD1d for 24 h, respectively. Then, the cells were infected with DiD-ASFV at 37°C. After 1 h, the cells were washed and collected for ASFV internalization by flow cytometry analysis. (F and G) PAMs were transfected with scrambled siRNA (si-Ctrl) and si-CD1d for 24 h, respectively. Then, cells were infected with DiD-ASFV at 4°C for 1 h, washed of free DiD-ASFV, and then transferred to 37°C to allow incorporation of DiD-ASFV up to 1 h. The cells were observed using high-magnification confocal microscopy for DiD-ASFV internalization after 1 h of incubation at 37°C. White arrows show the internalized DiD-ASFV. Bar = 10 μm (F). Statistical analysis of DiD-ASFV internalization. To quantify the binding and internalization of DiD-ASFV, WGA-labelled cell membranes (Grey) were used as a reference, where colocalized (Red) with the cell membrane was the binding virus, and internal (Red) was the internalized virus. More than two hundred cells were counted per sample, and the percentage of cells that internalized DiD-ASFV positive was calculated. (G). The data were analysed statistically using one-way analysis of variance (ANOVA). The results are presented as the mean ± standard deviation of three independent measurements. ****P < 0.0001; ***P < 0.001; **P < 0.01.

It has been reported that a commercially available cell line, MA104, could be used as a substitute for PAMs, which can be used for the isolation of ASFV strains and studied the ASFV infection-related biological processes [38,39]. The homology of the porcine C*D1d* gene (NCBI Gene ID:103223798) and African green monkeys *CD1d* gene (NCBI Gene ID: 100124526) was 79.2%, showing a high homology (Supplementary Figure 2(A)). To further confirm that CD1d is required for ASFV infection, the CRISPR/Cas9 system was used to knockout the *CD1d* gene in MA104 cells. Two independent clonal lines (MA104-ΔCD1d-1 and MA104-ΔCD1d-2) were selected based on the sequencing results (Supplementary Figure 2(B)) and the expression levels of CD1d in the two MA104-ΔCD1d cell lines were detected by flow cytometry analysis (Supplementary Figure 2(C)). To accurately evaluate the effect of CD1d on ASFV infection, we re-expressed porcine CD1d in these knockout cell lines by lentivirus transduction (Supplementary Figure 2(D)). Then the cells were infected with rASFV-Gluc-GFP. We found that deletion of the *CD1d* gene significantly decreased the virus infection, genomic DNA copies and luciferase reporter gene expression level (Figure 3(A–C)). However, re-expression of CD1d in the knockout cell lines rescued viral infection by increasing the genomic copies and luciferase reporter gene expression (Figure 3(A–C)), indicating that the ASFV infection defect was mainly caused by the deficiency of CD1d expression. Taken together, these results demonstrate that CD1d is a key host factor required for ASFV infection in MA104 cells.

It is well-known that ASFV virions adhere and subsequently internalize into the targeting cells, which eventually leads to the release of viral genomes to the cytoplasm for viral replication. We next investigated which step in ASFV entry is affected by the depletion of CD1d. As shown in Figure 3(D), attached virions were not decreased during ASFV entry, but internalized virions were significantly reduced in the knockout of CD1d. To further confirm the role of CD1d, ASFV-susceptible PAMs were chosen to validate the results by knockdown of CD1d expression. Flow cytometry analysis results showed that endocytosis of DiD-ASFV virions was inhibited after the knockdown of CD1d expression (Figure 3(E)). Furthermore, using high magnification confocal microscopy, we also observed the internalization of DiD-ASFV virions at 60 min post-ASFV infection (Figure 3(F)). In PAMs transfected with scrambled siRNA (si-Ctrl), DiD-ASFV virion was internalized into the cells and formation speckled patterns are increased (Figure 3(F,G)). In contrast, PAMs transfected with si-CD1d showed a much lower frequency of ASFV internalization and the quantified number of cells showed that ASFV internalization was significantly decreased (Figure 3(G)). Furthermore, flow cytometry analysis results showed that CD1d expression on the membrane surface of PAMs and MA104 cells gradually decreased during the ASFV virions internalization (Supplementary Figure 2(E)). These results suggest that CD1d plays a significant role in the internalization of ASFV virions.

### CD1d facilitates ASFV internalization via CME

Previous studies reported that ASFV virions can be internalized into the targeting cells via CME and macropinocytosis pathways [40,41]. To verify which internalization pathway could be affected by CD1d during ASFV infection, PAMs were transfected with si-Ctrl or si-CD1d and a confocal microscopy assay was performed to test the changes of Cy3-labelled transferrin (Cy3-Tf) or Alexa Fluor-594-labelled dextran (594-Dx). Transferrin is a marker for monitoring internalization through CME [42], and dextran is a liquid-phase marker that is widely used to label the internalization process via macropinocytosis [43]. The results showed that knockdown of CD1d expression significantly blocked the internalization of Cy3-labelled transferrin, but not the internalization of 594-labelled dextran, suggesting that CD1d may be mainly involved in the CME process (Supplementary Figure 3(A-D)).

**Figure 4.**
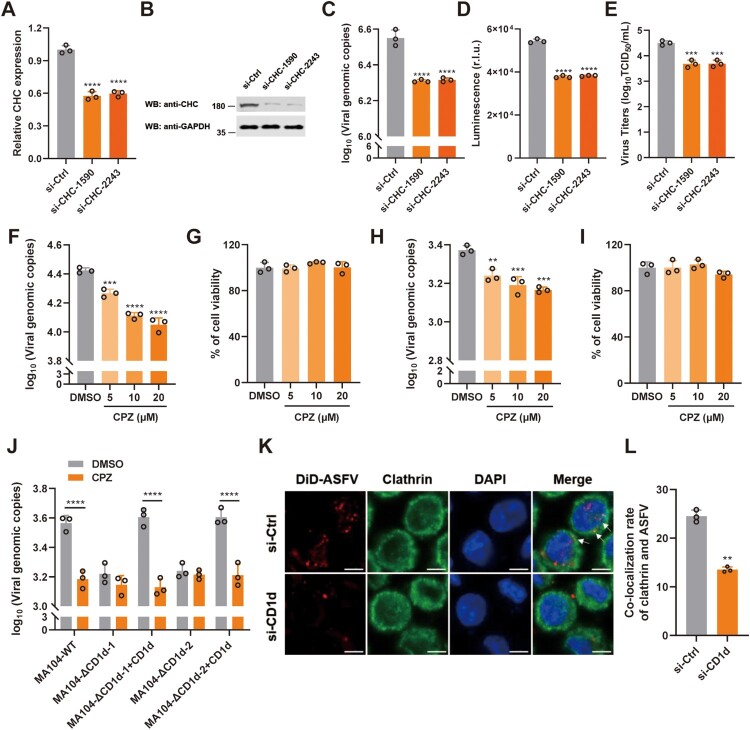
CD1d facilitates ASFV internalization via CME. (A–E) PAMs were transfected with scrambled siRNA control (si-Ctrl) and siRNAs targeting CHC (si-CHC) for 24 h, respectively. Then, the cells were infected with rASFV-Gluc-GFP at 37°C for 24 h, the mRNA level of CHC was examined by qPCR (A) and the protein level of CHC was examined by Western blot (B). The genomic level of ASFV in the cells was determined by qPCR (C) and luciferase activity (D), and the ASFV titre in the culture supernatant was determined by TCID_50_ assay (E). (F–I) PAMs (F) or MA104 (H) cells were incubated with different concentrations of CPZ for 30 min and then infected with ASFV. The genomic DNA copies of the ASFV in the cells was determined by qPCR. Cell viability of PAMs (G) or MA104 (I) cells was determined by WST-1 cell cytotoxicity assay kit (Beyotime). (J) MA104-WT and MA104-ΔCD1d cells were pretreated with CPZ (20 μM) for 30 min, then infected with ASFV HLJ/2018 at an MOI of 1 for 1 h at 4°C. Next, the cells were washed to remove any unbound ASFV and incubate at 37°C for 1 h. Viral genome level was detected by qPCR. DMSO was used as a control. The data were analysed statistically using one-way analysis of variance (ANOVA). (K and L) PAMs were transfected with si-Ctrl and si-CD1d for 24 h, respectively. Then, the cells were infected with DiD-ASFV at 37°C for 1 h. Next, the cells were washed with 0.2 M glycine (pH = 3) to remove any uninternalized ASFV. Immunofluorescence was performed to detect the co-localization of DiD-ASFV and clathrin. White arrows show the co-localized DiD-ASFV and clathrin. Bar = 5 μm (J). Statistical analysis of co-localization of DiD-ASFV and clathrin. More than two hundred cells were counted under fluorescence microscopy and cells showing co-localization were recorded (K). The data were analysed statistically using two-tailed Student’s *t*-tests. The results are presented as the mean ± standard deviation of three independent measurements. ****P < 0.0001; ***P < 0.001; **P < 0.01.

Previous study demonstrated that ASFV infection depends on the acidification of endosomes and the activity of dynamin GTPase, which are hallmarks of CME [44]. To verify whether the entry of ASFV virions into PAMs depends on CME, PAMs transfected with siRNAs to reduce clathrin heavy chain (CHC) expression and then the cells were infected with rASFV-Gluc-GFP. As expected, viral genome level and luciferase activity in ASFV-infected PAMs and virus titre in the culture supernatant were all significantly reduced after CHC knockdown (Figure 4(A–E)), suggesting that clathrin plays a pivotal role in mediating ASFV infection. As an inhibitor of CME, chlorpromazine (CPZ) can prevent the formation of coated pits at the plasma membrane and the assembly of clathrin lattices on endosomal membranes [45]. To further evaluate the function of CME during ASFV infection, we pretreated the PAMs and MA104 cells with 5, 10, and 20 μM of CPZ, and then analysed the transferrin internalization and ASFV infection. We found that 5 μM CPZ significantly reduced Cy3-Tf internalization, while 20 μM CPZ treatment completely abolished Cy3-Tf uptake in both cell types (Supplementary Figure 4(A, B)). Similarly, we demonstrated that CPZ treatment significantly blocked ASFV infection in a dose-dependent manner without obviously affecting cell viability in both cell types (Figure 4(F-I)). These results indicate that CME is a key pathway for ASFV virions to infect PAMs and MA104 cells.

To test whether CME is involved in CD1d-mediated ASFV entry, MA104-WT and MA104-ΔCD1d cells were treated with CPZ inhibitor at a concentration of 20 μM. We found that knockout of CD1d expression and CPZ treatment had a similar effect on ASFV internalization, suggesting that both CD1d and CME can mediate ASFV internalization. Interestingly, both knockout of CD1d expression and CPZ treatment did not show a synergistically inhibitory effect on ASFV infection, indicating CD1d may regulate ASFV internalization via CME. However, although the re-expression of CD1d in the MA104-ΔCD1d cells restored the cellular susceptibility to ASFV infection, CPZ treatment still strongly blocked the ASFV infection despite the re-expression of CD1d (Figure 4(J)), further supporting the concept that CME is involved in CD1d-mediated viral internalization. To further support our observation, we examined the co-localization of ASFV virions and clathrin in PAMs transfected with si-Ctrl or si-CD1d. We found that ASFV virions was co-localized with clathrin, a key component of CME in ASFV-infected PAMs. The co-localization of internalization virions with clathrin was significantly reduced in the CD1d-deficient PAMs (Figure 4(K, L)). Taken together, the results suggest that CD1d facilitates the internalization of ASFV virions via CME.

### The interaction of CD1d and EPS15 is involved in ASFV internalization

**Figure 5.**
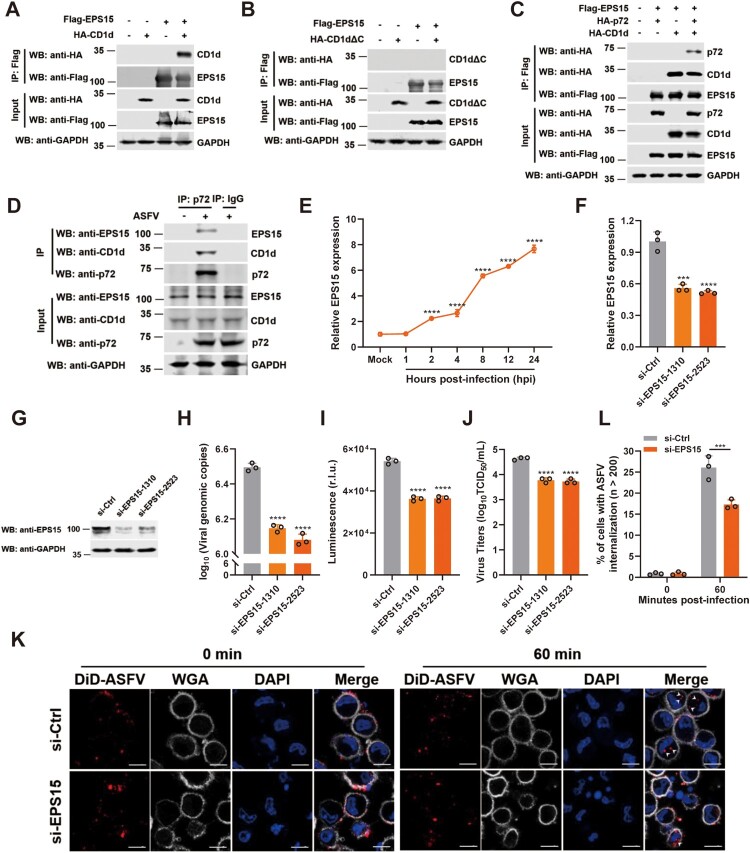
EPS15 interacts with CD1d and is involved in ASFV internalization via CME. (A-B) The cell lysates from HEK293T cells overexpressing Flag-EPS15 and HA-CD1d (A) or HA-CD1dΔC (B) were subjected to immunoprecipitation with anti-Flag antibody. Immunoprecipitants and whole-cell lysates (Input) were immunoblotted with an anti-Flag and anti-HA antibodies, respectively. (C) The cell lysates from HEK293T cells overexpressing Flag-EPS15, HA-p72, and HA-CD1d were subjected to immunoprecipitation with anti-Flag antibody. Immunoprecipitants and whole-cell lysates (Input) were immunoblotted with an anti-Flag and anti-HA antibodies, respectively. (D) PAMs were infected with ASFV HLJ/2018 at an MOI of 5 or mock-infected for 48 h, and the cell lysates were subjected to immunoprecipitation with anti-p72 antibody. Immunoprecipitants and whole-cell lysates (Input) were immunoblotted with anti-p72, anti-CD1d, and anti-EPS15 antibodies, respectively. (E) PAMs were mock-infected or infected with ASFV for 1, 2, 4, 8, 12 and 24 h, and the mRNA levels of EPS15 in each time point in PAMs were analysed by qRT-PCR. (F-J) PAMs were transfected with si-Ctrl and si-EPS15s for 24 h, respectively. Then, the cells were infected with ASFV at 37°C for 24 h, and the mRNA level of EPS15 was examined by qPCR (F) and the protein level of EPS15 was examined by Western blot (G). The genome level of the ASFV in cells was determined by qPCR (H), luciferase activity in cells was measured (I), and the virus titre in the culture supernatant was determined by TCID_50_ assay (J). (K and L) PAMs were transfected with si-Ctrl and si-EPS15 for 24 h, respectively. Then, cells were infected with DiD-ASFV at 4°C for 1 h, washed away unbound DiD-ASFV, and then transferred to 37°C to allow incorporation of DiD-ASFV for up to 60 min. The cells were observed using high-magnification confocal microscopy for DiD-ASFV internalization. White arrows show the internalized DiD-ASFV. Bar = 10 μm (K). Statistical analysis of DiD-ASFV internalization. More than two hundred cells were counted under fluorescence microscopy (L). The data were analysed statistically using one-way analysis of variance (ANOVA). The results are presented as the mean ± standard deviation of three independent measurements. ****P < 0.0001; ***P < 0.001.

EPS15 has been reported to be involved in the internalization of ASFV virions into Vero and WSL cells through CME [40]. To test whether EPS15 is associated with CD1d and promotes ASFV virion internalization, a Co-IP assay was first performed. We found that EPS15 interacted with full-length CD1d but not the extracellular domain of CD1d, suggesting that EPS15 may interact with the intracellular domain of CD1d (Figure 5(A, B)). We further explored the association of p72, CD1d and EPS15 by Co-IP assay, and found that the three proteins formed a complex where CD1d is necessary for the formation of the p72-CD1d-EPS15 complex (Figure 5(C)). To confirm the association of these endogenous proteins in PAMs, the cells were infected with ASFV-WT and then Co-IP was performed. We found that the three proteins also formed a complex during ASFV infection (Figure 5(D)). These results suggest that CD1d may act as a mediator protein between p72 and EPS15 to regulate the internalization of ASFV virions.

To further verify whether EPS15 is required for ASFV infection in PAMs, the expression pattern of EPS15 was first investigated during virus infection. We found that the mRNA level of EPS15 was upregulated in PAMs infected with ASFV-WT (Figure 5(E)). To test whether EPS15 promotes viral infection and replication, PAMs were transfected with siRNAs to knockdown EPS15 expression and then infected with rASFV-Gluc-GFP. We found that deficiency of EPS15 dramatically decreased the viral genomic DNA level, luciferase activity in cells, and virus titre in the culture supernatant (Figure 5(F–J)). Furthermore, we observed that ASFV virions internalization obviously decreased in EPS15-deficient PAMs (Figure 5(K, L)). Taken together, these results suggest that EPS15 plays a pivotal role in supporting ASFV internalization during viral infection in PAMs.

**Figure 6.**
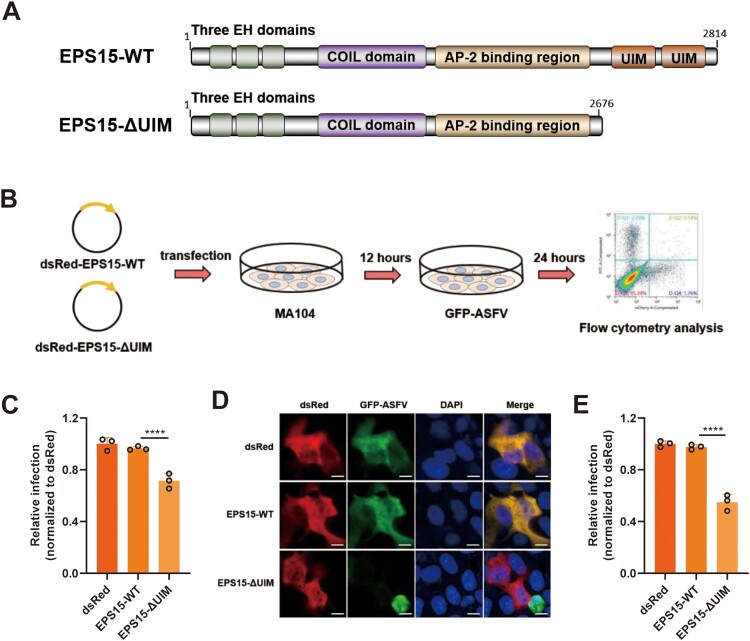
The UIM domains of EPS15 are required for ASFV internalization. (A) The diagram shows functional regions of the porcine EPS15-WT and EPS15ΔUIM. UIM, ubiquitin-interacting domain. (B) The concise procedure for sample preparation and treatment. (C–E) MA104 cells were overexpressed with dsRed, dsRed-EPS15-WT and dsRed-EPS15-ΔUIM, respectively. The transfected cells were then infected with rASFV-Gluc-GFP (GFP-ASFV). At 24 hpi, the cells were analysed by flow cytometry (C), observed by fluorescence microscopy (D) (Bar = 10 μm), and manually calculated for viral infection rate (E). More than two hundred transfected cells were examined in each case. The data were analysed statistically using one-way analysis of variance (ANOVA). The results are presented as the mean ± standard deviation of three independent measurements. ****P < 0.0001; ***P < 0.001.

Porcine EPS15 contains four domains: an N-terminal domain with three EH motifs, a central domain involved in dimerization, and a C-terminal regulatory domain with the AP-2 binding site followed by two ubiquitin-interacting motifs (UIMs) (Figure 6(A)) [46]. Previous studies showed that the UIMs of EPS15 play important roles in endocytosis and endosomal transport [47,48]. To examine whether the UIMs domain of EPS15 plays a key role in ASFV infection, EPS15 or EPS15-ΔUIM was transiently overexpressed in MA104 cells which were then infected with rASFV-Gluc-GFP (Figure 6(B)). We found that the level of ASFV infection was significantly reduced in cells overexpressing EPS15-ΔUIM compared to the cells overexpressing EPS15 (Figure 6(C–E)). These results suggest that ASFV infection may require intact EPS15 in MA104 cells, illustrating the importance of EPS15 for ASFV infection.

## Discussion

ASFV infection causes a highly contagious and acute hemorrhagic viral disease, resulting in huge economic losses to the swine industry worldwide. There is no vaccine or drug to effectively control ASF, mainly because our understanding of the virus is still limited, especially on the molecular mechanisms of entry and replication. For the entry of ASFV, the inner envelope component pE248R has been proven to be essential for membrane fusion and cytoplasmic core delivery [49]. And another study revealed that the inner envelope protein pE199L was also required for membrane fusion and core penetration [50]. Recently, the host endosomal proteins Nieman-Pick C has been proven to promote membrane fusion and release viral nucleic acid by interacting with viral proteins E199L and E248R after the virus enters the target cells [26]. These are all the late events in the entry process of the virus. For early events, it is not known which molecules are involved in the attachment and internalization of ASFV. In this study, we found that ASFV virions bound to the host factor CD1d on the cell membrane via capsid protein p72. Then CD1d interacts with EPS15 and promotes ASFV virions internalization into the host cells via CME. We reveal an early event of ASFV invasion, the triggering of viral adhesion and internalization, which is important for subsequent capsid disassembly and naked core release of the virus in the endosomal pathway.

In the early stages of viral infection, structural proteins of virions usually interact with host membrane receptors or co-receptors, which then mediate viral internalization into the host cells. Previous reports demonstrated that ASFV virions without external lipid envelopes can also infect cells [4,9]. As one of the main capsid proteins of ASFV virions, p72 encoded by the *B646L* gene may contribute to viral entry. We found that the anti-p72 antibody could effectively block the viral infection while the antibodies against p34 and pB318L failed to block viral infection. These results suggest that p72 localizes on the surface of the ASFV virions and is involved in viral infection by potentially binding to the host membrane receptors or co-receptors. Here, the host membrane factor CD1d was found by IP-MS screen and interacted with p72 during the ASFV infection. Importantly, the knockdown of CD1d expression, blocking the host cells with anti-CD1d antibody, and pre-incubation of the virions with recombinant CD1d proteins all dramatically blocked ASFV infection, highlighting its critical role in viral entry. In addition, we also found that CD1d is involved in herpes simplex virus-1 (HSV-1), pseudorabies virus (PRV) and porcine reproductive and respiratory syndrome virus (PRRSV) infections, suggesting that CD1d is a pan-host factor involved in viral infection, although the mechanism needs further investigation (Supplementary Figure 5(A–D)).

CD1 family members represent a unique lineage of antigen-presenting molecules related to MHC class I proteins with limited sequence similarity and domain organization. CD1 family in pigs has four members: CD1a, CD1b, CD1d, and CD1e, consisting of α1, α2, and α3 domains, which are encoded by different genes [51]. We analysed the homology of CD1a, CD1b, CD1d, and CD1e amino acid sequences by the Clustal W method in MegAlign software. The amino acid homology of CD1d (GenBank accession number: XP_005663291.1) to CD1a (GenBank accession number: NP_998996.1), CD1b (GenBank accession number: NP_001090961.1) and CD1e (GenBank accession number: NP_001090960.1) were 47.8%, 50.8% and 53.1%, respectively, indicating that the amino acid sequences between CD1d and the other three members were rather different (Supplementary Figure 6(A)). Among these members, CD1e was almost not expressed in PAMs, and CD1a and CD1b seemed to have no obvious effect on ASFV infection (Supplementary Figure 6(B–G)).

Monocyte-macrophages are the main target cells of ASFV [33]. In addition, a commercial cell line MA104 has been reported to study the biological process of ASFV infection [38,52,53]. Although the infection rate of MA104 cells with the current ASFV strain in China was relatively low [54,55], this is the only cell line that can be found to study viral invasion and replication. Therefore, we used this cell line to construct CD1d-edited cells. Our results showed that the CD1d-edited cell lines of MA104 had only a partial effect on the infectivity of ASFV, indicating that CD1d was only one of the host factors affecting ASFV entry, and other host factors may be involved in the viral invasion.

The attachment and internalization of ASFV mainly depend on the host factors on the membrane of target cells. Through antibody-blocking assay, we demonstrated that CD1d could function as a surface factor to mediate ASFV entry. The subsequent direct interaction between p72 and CD1d also has been proved. Interestingly, we found that CD1d did not affect the attachment of the virus but affected its internalization (Figure 3(F)), suggesting that besides CD1d, other host factors can mediate the attachment of ASFV. While other host factors may promote the internalization process after facilitating the viral attachment [56,57], so knockdown of CD1d expression clearly affects the viral internalization. The deletion of CD1d also significantly affected the internalization of the ASFV, indicating that CD1d played an important role in the internalization process of ASFV. In addition, we also observed that CME and clathrin expression was needed for ASFV entry by CPZ inhibitor treatment and siRNA interference. Taken together, our findings show that CD1d affects the internalization of ASFV in PAMs and MA104 cells via CME.

CME is the uptake of cargo molecules from the cell membrane into the cytoplasm through clathrin-coated vesicles (CCVs). The viral infection-mediated CME involves several steps, including viral attachment to the host cell membrane receptors, activation of signalling cascades, local clathrin assembly, virus-attached membrane invagination, maturation. and fission of CCVs [58]. During viral endocytosis, clathrin does not directly bind to cargo receptors, whereas it relies on adaptor proteins (such as EPS15, AP180, AP-2, and Epsin1) to be recruited to the cytoplasm membrane [21]. In this study, we found that p72 interacts with CD1d and CD1d further recruits EPS15 during ASFV infection. The three proteins form a complex during ASFV infection, and CD1d is required for maintaining the stability of the complex. In agreement with our model, we found that EPS15 and CD1d have similar functions during ASFV infection. Expression of EPS15 is enhanced in response to ASFV infection, and loss of EPS15 dramatically blocked ASFV internalization and thus ASFV infection. Our model suggests that CD1d and EPS15 cooperate to promote the internalization of ASFV virions via CME. As a key protein in the formation of CCVs, AP-2 provides a binding site for EPS15 and clathrin, forming the EPS15-AP-2-clathrin complex which leads to clathrin nucleation [22]. In this study, we also tested the roles of AP-2, AP180 and Epsin1 in ASFV infection in PAMs and found that the AP-2 complex, but not AP180 and Epsin1, also promoted ASFV infection (Supplementary Figure 7(A–Y)).

**Figure 7.**
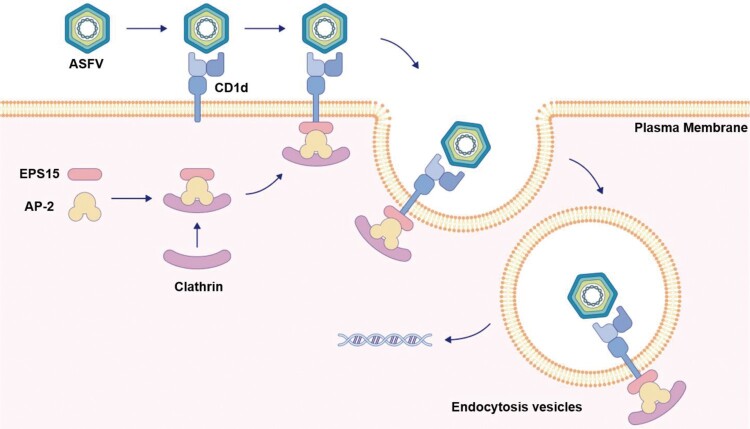
Schematic illustration of CD1d in enhancing ASFV endocytosis as a host factor. ASFV virions enter host cells via host factor CD1d on the cell membrane. The p72 on the surface of the ASFV virion binds with CD1d on the cell membrane. The signal is then transmitted to EPS15 in the cytoplasm to form the p72-CD1d-EPS15 axis, where CD1d recruits EPS15 through the interaction between CD1d and EPS15 intracellular domains. The process promotes the formation of EPS15-AP-2-clathrin complexes and aggregation, resulting in ASFV virions endocytosis during ASFV infection.

Overall, our study identified that the capsid protein p72 on the ASFV virions binds to the host membrane factor CD1d, which further recruits EPS15 to form the p72-CD1d-EPS15 complex, finally leading to endocytosis of ASFV virions during ASFV infection (Figure 7). Our findings shed new light on the molecular mechanism of ASFV infection and provide a new perspective for the development of antiviral therapies.

## Author contributions

X.C., J.Z., C.W. and Z.B. designed research; X.C., J.Z. and C.L. performed research; T.L., X.W., X.L., M.B., J.L., L.H. Z.Z., and Z.B. contributed new reagents/analytic tools; X.C., C.W., J.Z., C.L. and T.L. analysed data; X.C., J.Z. and C.W. wrote the paper.

## Supplementary Material

Supplemental Material
