# Supplementary material for: CD1d facilitates African swine fever virus entry into the host cells via clathrin-mediated endocytosis
Source: Emerg Microbes Infect. 2023 Jun 22;12(2):2220575. doi: 10.1080/22221751.2023.2220575 (PMC10288936; doi:10.1080/22221751.2023.2220575)
Supplement: Supplemental Material [file TEMI_A_2220575_SM7641.docx]

**Supplementary data**

**CD1d facilitates African swine fever virus entry into the host cells** **via clathrin-mediated endocytosis**

Xin Chen^1, #^, Jun Zheng^1, 2, #^, Chuanxia Liu^1, #^, Tingting Li^1^, Xiao Wang^1^, Xuewen Li^1^, Miaofei Bao^1^, Jiangnan Li^1, 2^, Li Huang^1, 2^, Zhaoxia Zhang^1, 2^, Zhigao Bu^1^, Changjiang Weng^1, 2,^ *

^1^ Division of Fundamental Immunology, National African Swine Fever Para-reference Laboratory, State Key Laboratory for Animal Disease Control and Prevention, Harbin Veterinary Research Institute, Chinese Academy of Agricultural Sciences (CAAS), Harbin 150069, China

^2^ Heilongjiang Provincial Key Laboratory of Veterinary Immunology, Harbin 150069, China

^#^ These authors contributed equally to this work.

*Correspondence and requests for materials should be addressed to C.W. [(wengchangjiang@caas.cn](mailto:(wengchangjiang@caas.cn;)).

Changjiang Weng, PhD. Professor,

State Key Laboratory for Animal Disease Control and Prevention, Harbin Veterinary Research Institute, Chinese Academy of Agricultural Sciences (CAAS), Harbin 150069, China

Email: [wengchangjiang@caas.cn](mailto:Wengcj@caas.cn)


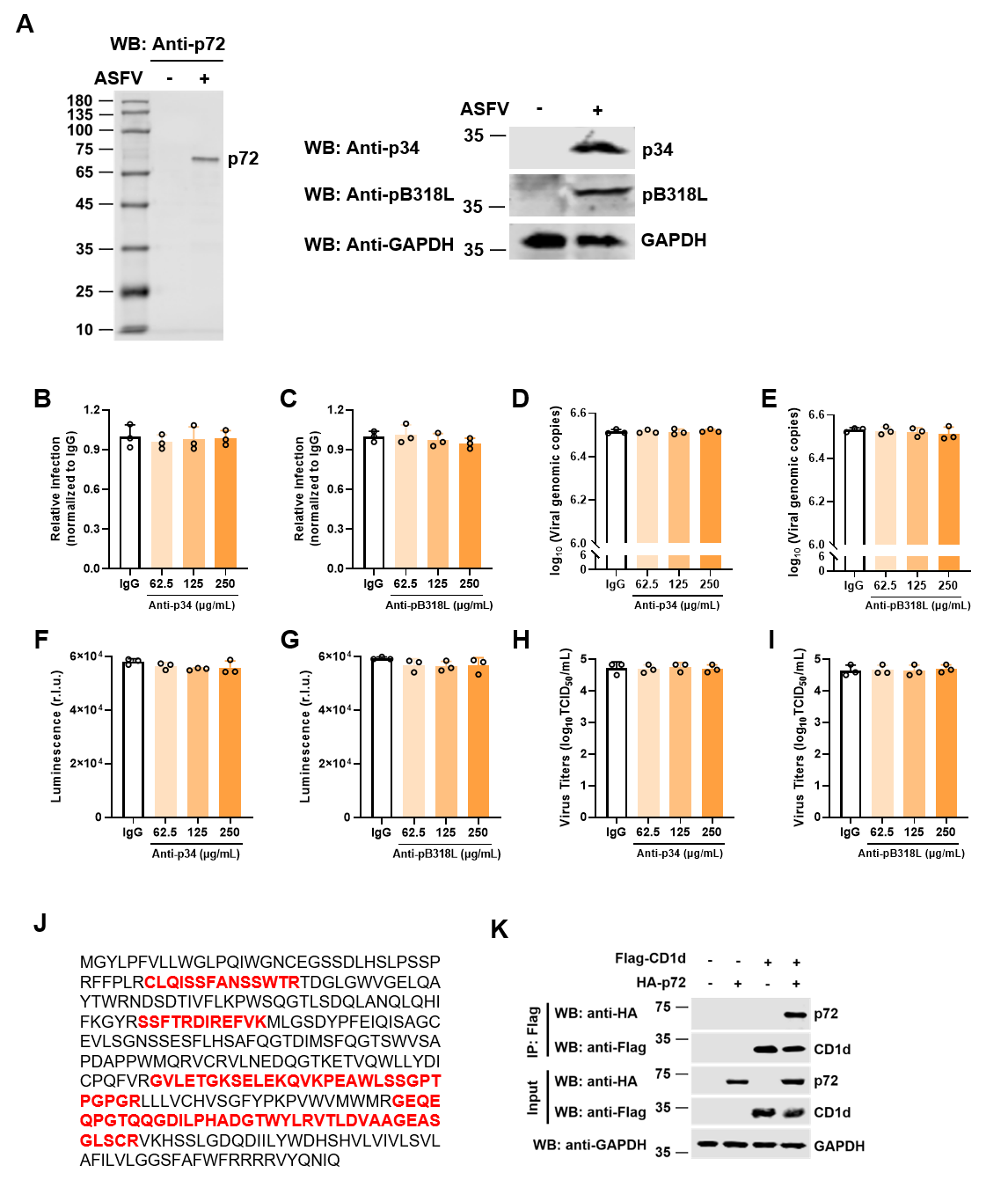


# Figure S1. Screening of ASFV p72-interacting proteins.

**(A)** PAMs were infected with ASFV-WT for 24 h. After cell lysis, p72, p34 and pB318L of ASFV were detected by Western blotting. Anti-GAPDH were used as a control. **(B-I)** As controls, the rASFV-Gluc-GFP virions were incubated with anti-p34 and anti-pB318L antibodies at the indicated concentrations for 4 h at 4 °C. Then, the virus and antibody mixtures were used to infect PAMs for 24 h. The infection ratio of ASFV was determined by flow cytometry analysis (A and E). qPCR (B and F) and luciferase activity (C and G) was used to detect the genomic DNA level of the virus in cells. The virus titer in the culture supernatant was determined by TCID_50_ assay (D and H). The data were analyzed statistically using one-way analysis of variance (ANOVA). The results are presented as mean ± standard deviation of three independent measurements. **(J)** Identiﬁcation of CD1d by LC-MS/MS analysis. Peptides matched to the CD1d amino acid sequence are highlighted in red. **(K)** HEK293T cells were transfected with plasmids expressing Flag-CD1d and HA-p72. The cell lysates were subjected to Co-IP with anti-Flag antibodies. Immunoprecipitants and whole-cell lysates (Input) were immunoblotted with anti-Flag, anti-HA and anti-GAPDH antibodies, respectively. ****, *P*< 0.0001; ***, *P*< 0.001; **, *P*< 0.01.


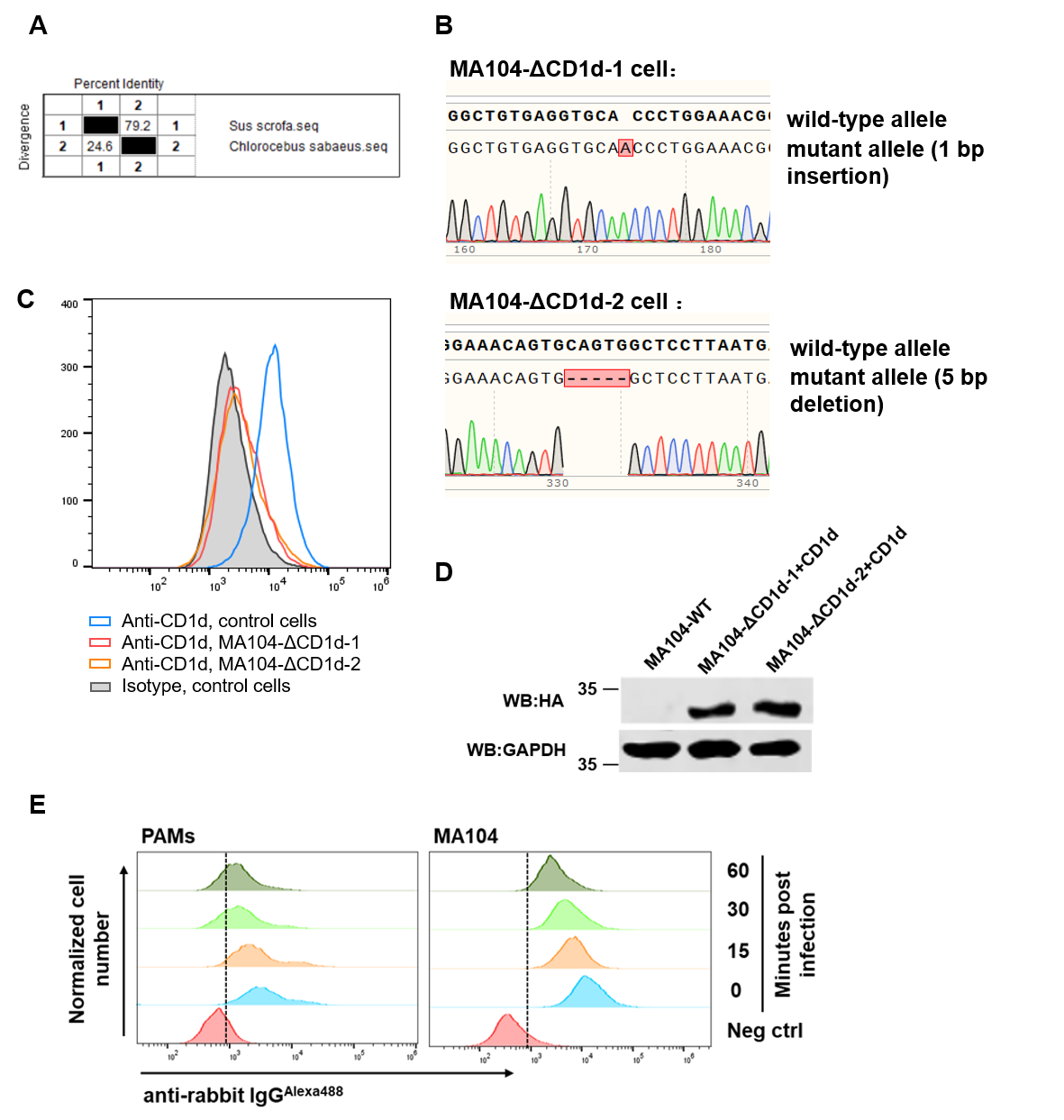


# Figure S2. Generation and identification of CD1d knockout cell lines and exogenously re-expressing CD1d in MA104-ΔCD1d cell lines.

**(A)** Nucleotide sequence homology of CD1d between pigs and African green monkeys was analyzed by the Clustal W method in MegAlign software. **(B)** Sequencing of CD1d in MA104-WT and MA104-ΔCD1d cell lines. Sequencing data shows an alignment and individual out-of-frame deletions. **(C)** The CD1d expression on the surface of MA104 cells and MA104-ΔCD1d cell lines were tested by flow cytometry using anti-CD1d antibodies and an isotype control polyclonal antibody. One representative experiment of two is shown. **(D)** MA104-ΔCD1d+CD1d stable cell lines re-expressing CD1d were examined by western blot. **(E)** PAMs and MA104 cells were infected at 37 ℃ with ASFV HLJ/18 at an MOI of 1. After 0, 15, 30 and 60 min, the CD1d expression on the surface of the cells were tested by flow cytometry using an anti-CD1d antibody and an isotype control polyclonal antibody respectively. One representative experiment of three is shown.


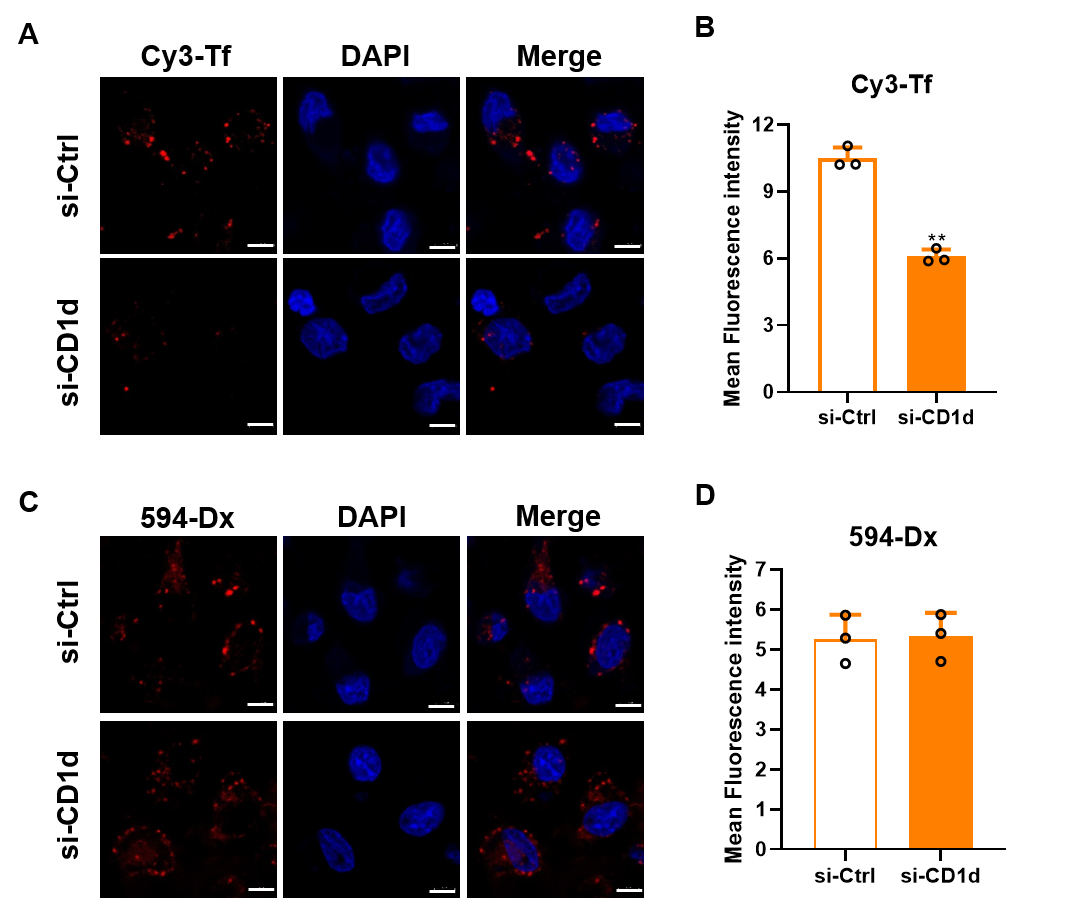


# Figure S3. CD1d is involved in the CME pathway, but not in macropinocytosis.

**(A-D)** PAMs were transfected with si-Ctrl and si-CD1d for 24 h, respectively. Then, the cells were incubated with Cy3-labeled transferrin or Alexa Fluor-594-labeled dextran and observed the transferrin (A) or dextran (C) internalization using high-magnification confocal microscopy. The mean transferrin (B) or dextran (D) fluorescence intensity of more than two hundred cells per condition in a single-matched experiment is shown. Experiments were repeated three times with similar phenotypes. Sample images corresponding to each condition are shown. The data were analyzed statistically using two-tailed Student’s t-tests. The results are presented as the mean ± standard deviation of three independent measurements. Bar = 5 μm. **, *P* < 0.01.


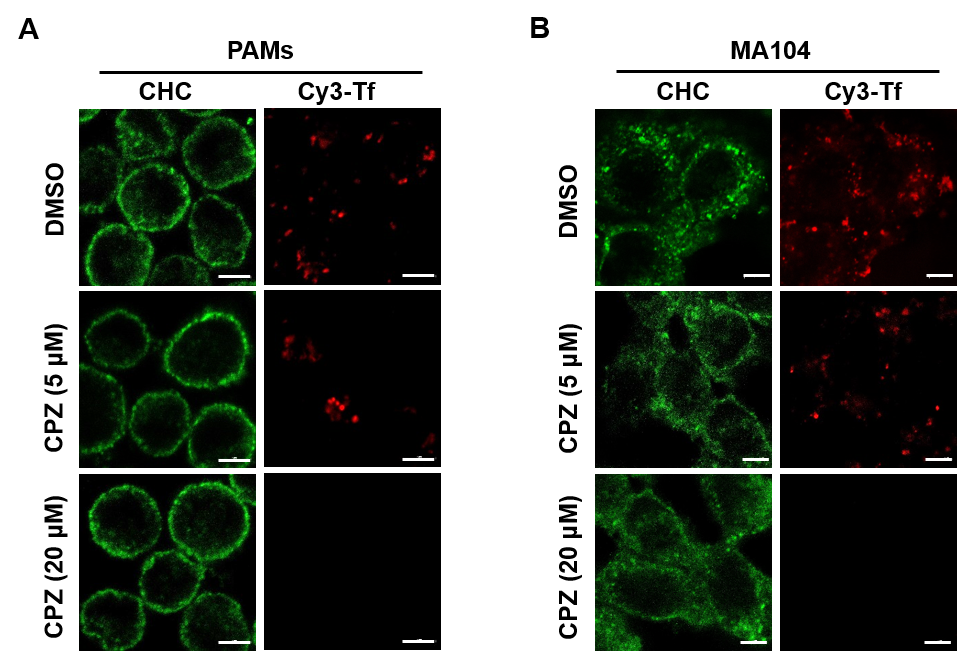


# Figure S4. Inhibition of Cy3-Tf internalization by CPZ.

**(A and B)** PAMs (A) and MA104 (B) cells were examined by confocal microscopy for Cy3- Tf uptake after treatment with different CPZ concentrations. The clathrin distribution was analyzed with a specific polyclonal antibody followed by anti-rabbit conjugated to Alexa Fluor 488. Bar = 5 μm.


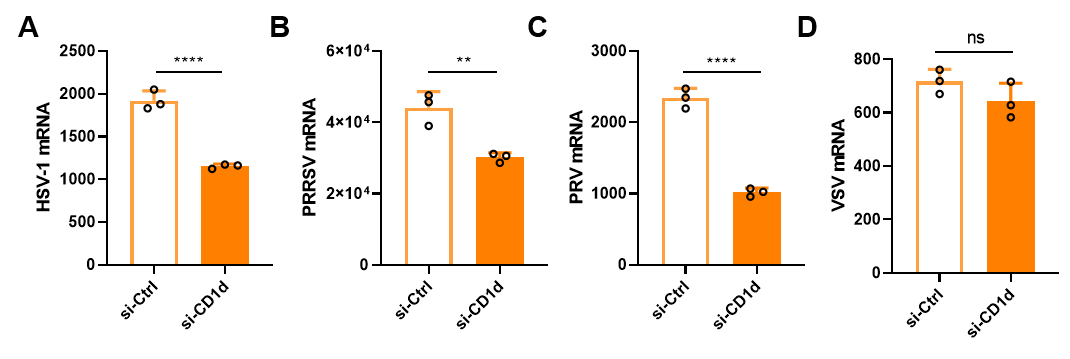


## Figure S5. The Effects of CD1d on other viral infection in PAMs.

**(A-E)** PAMs were transfected with scrambled siRNA (si-Ctrl) and siRNA targeting CD1d (si-CD1d) for 24 h, respectively. Then, the cells were infected with HSV-1 (A), PRRSV (B), PRV (C) and VSV (D) at 37 ℃ for 24 h. Viral replication was analyzed by qPCR. The data were analyzed statistically using two-tailed Student’s t-tests. The results are presented as the mean ± standard deviation of three independent measurements. ****, *P*< 0.0001; **, *P*< 0.01; ns, *P*> 0.05.


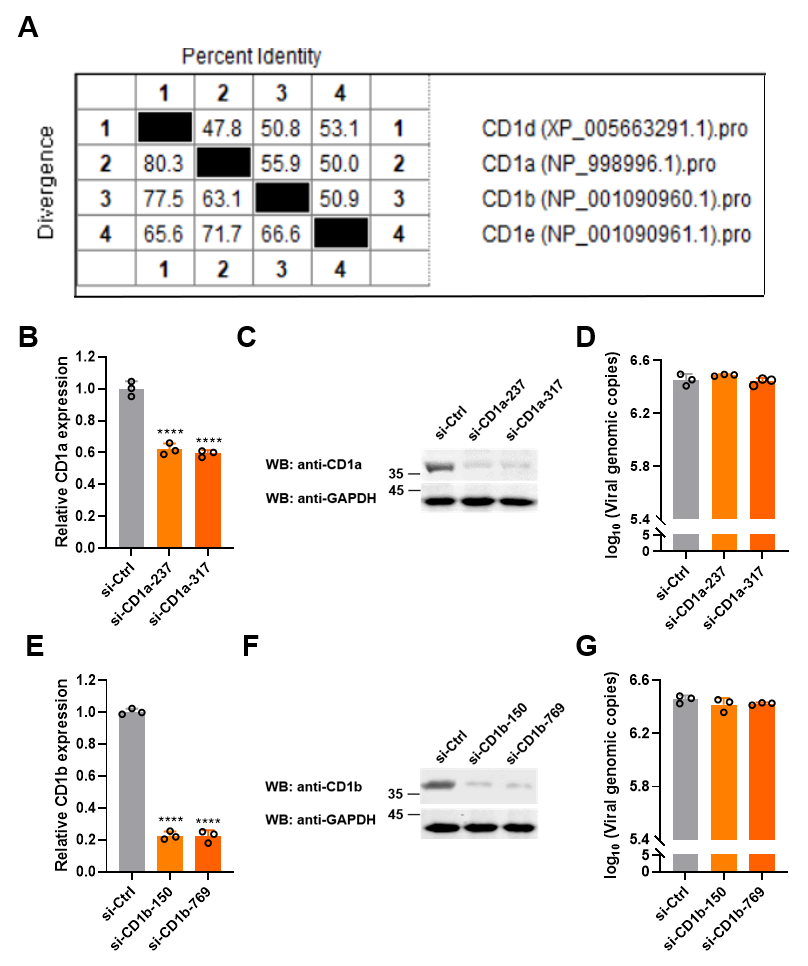


## Figure S6. Other CD1 family members did not affect ASFV infection.

**(A)** Amino acid sequence homology of CD1 family members was analyzed by the Clustal W method in MegAlign software. **(B-D)** PAMs were transfected with scrambled siRNA (si-Ctrl), and siRNAs targeting CD1a as indicated for 24 h, respectively. Then, cells were infected with ASFV-WT at 37 ℃ for 24 h, the mRNA levels of CD1a were examined by qRT-PCR (B) and the protein levels of CD1a were examined by Western blot (C). The genome level of the virus was determined by qPCR (D). **(E-G)** PAMs were transfected with scrambled siRNA (si-Ctrl), and siRNAs targeting CD1b as indicated for 24 h, respectively. Then, cells were infected with ASFV-WT at 37 ℃ for 24 h, and the mRNA levels of CD1b were examined by qRT-PCR (E) and the protein levels of CD1b were examined by Western blot (F). The genome level of the virus was determined by qPCR (G). The data were analyzed statistically using one-way analysis of variance (ANOVA). The results are presented as the mean ± standard deviation of three independent measurements. ****, *P*< 0.0001.


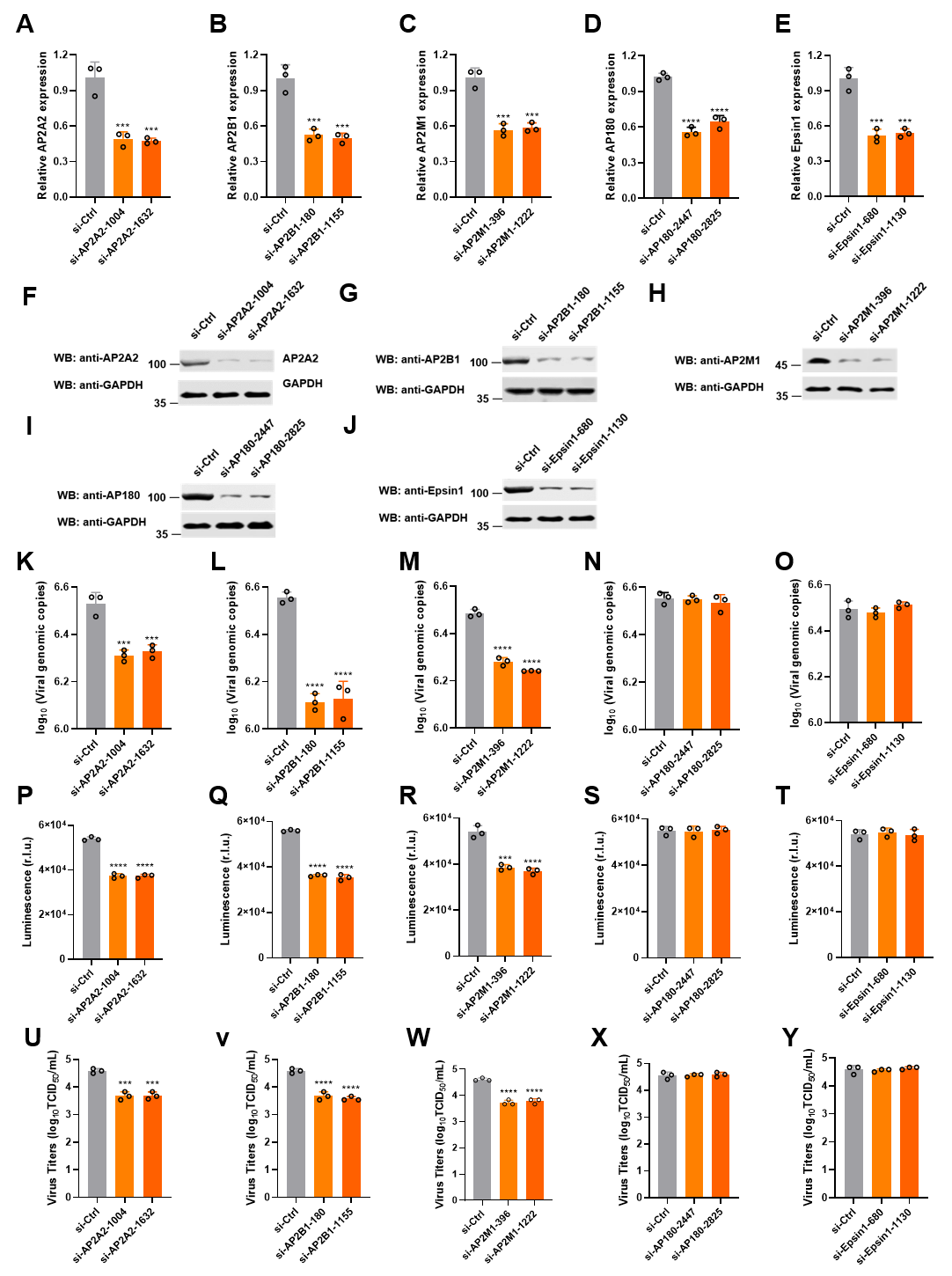


## Figure S7. Identification of adaptor proteins involved in ASFV infection.

**(A-Y)** PAMs were transfected with scrambled siRNA (si-Ctrl), siRNAs targeting AP2A2, AP2B1, AP2M1, AP180 and Epsin1 as indicated for 24 h, respectively. Then, cells were infected with ASFV at 37 ℃ for 24 h, the mRNA levels of AP2A2 (A), AP2B1 (B), AP2M1 (C), AP180 (D), and Epsin1 (E) were examined by qPCR and the protein levels of AP2A2 (F), AP2B1 (G), AP2M1 (H), AP180 (I), and Epsin1 (J) were examined by Western blot. The genome level of the virus was determined by qPCR (K-O) and luciferase activity (P-T), and the virus titer in the culture supernatant was determined by TCID_50_ assay (U-Y). The data were analyzed statistically using one-way analysis of variance (ANOVA). The results are presented as the mean ± standard deviation of three independent measurements. ****, *P*< 0.0001; ***, *P*< 0.001.

# Table S1. Identification of p72 binding partners by mass spectrometry.

| **Number** | **Mass** | | **Score** | **Matches** | **Sequences** | **emPAI** | **Coverage** | **Protein description** |
| --- | --- | --- | --- | --- | --- | --- | --- | --- |
| 1 | 38864 | 898 | | 104(43) | 9(5) | 1.09 | 28% | CD1d antigen |
| 2 | 38284 | 177 | | 9(5) | 4(3) | 0.28 | 11% | guanine nucleotide-binding protein subunit beta-4 |
| 3 | 33162 | 175 | | 18(7) | 6(4) | 0.61 | 17% | ADP/ATP translocase 2 |
| 4 | 35511 | 169 | | 13(6) | 8(3) | 0.31 | 27% | G-beta like protein |
| 5 | 33117 | 156 | | 12(5) | 6(4) | 0.47 | 17% | mitochondrial solute carrier family 25-member 6 |
| 6 | 35427 | 152 | | 12(5) | 7(5) | 0.56 | 23% | E3 ubiquitin-protein ligase CHIP |
| 7 | 36717 | 150 | | 19(6) | 5(2) | 0.19 | 13% | Golgi to ER traffic protein 4 homolog isoform X1 |
| 8 | 40420 | 142 | | 9(6) | 4(4) | 0.37 | 8% | solute carrier family 25 member 3 |
| 9 | 34115 | 103 | | 12(5) | 9(4) | 0.59 | 28% | cell division cycle 2 |
| 10 | 32086 | 101 | | 10(3) | 5(3) | 0.34 | 14% | voltage-dependent anion channel 2 |
| 11 | 34904 | 97 | | 12(4) | 9(3) | 0.31 | 24% | mitochondrial glutamate carrier 1 |
| 12 | 70340 | 95 | | 10(3) | 6(2) | 0.10 | 10% | heat shock protein 70.2 |
| 13 | 36283 | 84 | | 6(3) | 4(2) | 0.19 | 12% | ELAV (embryonic lethal, abnormal vision, Drosophila)-like 1 |
| 14 | 32237 | 82 | | 14(6) | 7(4) | 0.63 | 14% | serine/threonine-protein phosphatase PGAM5, mitochondrial isoform X1 |
| 15 | 30148 | 76 | | 8(3) | 7(3) | 0.52 | 23% | ribosomal protein L7a |
| 16 | 12823 | 72 | | 8(1) | 5(1) | 0.60 | 26% | histone H1.3-like protein, partial |
| 17 | 16212 | 66 | | 6(3) | 4(2) | 0.46 | 30% | beta-globin |
| 18 | 25433 | 65 | | 3(1) | 2(1) | 0.13 | 6% | SFRS2 |
| 19 | 36169 | 63 | | 3(1) | 3(1) | 0.19 | 9% | annexin A5 |
| 20 | 32352 | 61 | | 4(1) | 2(1) | 0.10 | 8% | malectin |
| 21 | 29102 | 54 | | 9(4) | 8(4) | 0.72 | 27% | proliferating cell nuclear antigen |
| 22 | 30529 | 50 | | 3(2) | 2(2) | 0.23 | 6% | acidic leucine-rich nuclear phosphoprotein 32 family member E |
| 23 | 26903 | 50 | | 3(1) | 3(1) | 0.42 | 13% | EF-hand domain-containing protein D1 isoform X1 |

# Table S2. The sequence of siRNA and primers used in this study.

| **siRNA** | **Sequence (5'-3')** |
| --- | --- |
| si-CD1d-245 | GCAAUGACUCGGACACCAUTT |
| si-CD1d-421 | GCUGGAUGUGAGGUGCUUUTT |
| si-CHC-1590 | GCAGUUUGCUCAAAUGUUATT |
| si-CHC-2243 | GCAGAGAAAGCAACUGUUATT |
| si-EPS15-1310 | GCUGCAGAUUUCUCUGCUATT |
| si-EPS15-2523 | GCACAUUGUCAAAGGUCAATT |
| si-CD1a-237 | GGAUCUGAUGGAAUCGGAATT |
| si-CD1a-317 | GGAAGCUUGAAUAUCCCUUTT |
| si-CD1b-150 | GGGUGACUUGCAGAUUCAUTT |
| si-CD1b-769 | GCAGAUGGGACGUGGUAUUTT |
| si-AP2A2-1004 | GCCUGGAGACGAUUCUGAATT |
| si-AP2A2-1632 | CCACGAGAACCUGGUCAAATT |
| si-AP2B1-180 | GGAAGGAGGCUGUGAAGAATT |
| si-AP2B1-1155 | UAGCAGAACUGAAGGAAUATT |
| si-AP2M1-396 | GCUGCCAUGGUCUUCGAAUTT |
| si-AP2M1-1222 | CCAUCGUGUGGAAGAUCAATT |
| si-AP180-2047 | CCAGCAAAGGUGGAUUCUUTT |
| si-AP180-2825 | CCAAGAAACCUCCAGCAAATT |
| si-Epsin1-680 | GCAGACGCUCAAGGACUUUTT |
| si-Epsin1-1130 | GGAGUCAUCCCUCAUGGAUTT |
| si-Ctrl | UUCUCCGAACGUGUCACGUTT |
| **Primer** | **Sequence (5'-3')** |
| **qPCR** |  |
| ASFV-p72-F | CTGCTCATGGTATCAATCTTATCGA |
| ASFV-p72-R | GATACCACAAGATCAGCCGT |
| HPRT-F | GCCGAGGATTTGGAAAAGG |
| HPRT-R | GCACACAGAGGGCTACGATG |
| ASFV-probe | FAM-CCACGGGAGGAATACCAACCCAGTG-TAMRA |
| Forward-CD1d | CACTCAGCATTTCAAGGAACAGACATC |
| Reverse-CD1d | TCCTCGTTGAGCACTCTACAGACC |
| Forward-CHC | AACCCAGCAAACATTGGCTTC |
| Reverse-CHC | CAATAATTACCACCTGGGCCTG |
| Forward-EPS15 | CACCACCACCTGGGAAAAGA |
| Reverse-EPS15 | TTGGGGCTATCATTGCCTGG |
| Forward-AP2A2 | CATCTCGGACATCCGCAACT |
| Reverse-AP2A2 | GAGCCTTGTCACCTTTAAATTTTG |
| Forward-AP2B1 | CATGCAGATGGGAGCAGTCG |
| Reverse-AP2B1 | CTGTCCCACTGCCGGACTTC |
| Forward-AP2M1 | TCTCCCGGGTCTACCGAGAT |
| Reverse-AP2M1 | CGAGCGATGTTGGTGACGG |
| Forward-AP180 | AAGGATCTGGTGCTCCCTCT |
| Reverse-AP180 | GGTGGGGATGTGTCAACAGT |
| Forward-Epsin1 | GCCCTTAGTTTGAGCCGTGA |
| Reverse-Epsin1 | GTCTCCCTCCTGCTCTCCTC |
| Forward-CD1a | CATCTGGCTTGAGTTGCCGA |
| Reverse-CD1a | CTGCCAATAAGATCCAGCCCA |
| Forward-CD1b | CCACCTCCATCACCTTGATAGTT |
| Reverse-CD1b | ACTCTGATATGACCAGCGCC |
| HSV-F | TGGGACACATGCCTTCTTGG |
| HSV-R | ACCCTTAGTCAGACTCTGTTACTTACCC |
| EMCV-F | TGAGCTTAGACCGATAGA |
| EMCV-R | GATGCAAACTTTCCCAAC |
| PRRSV-F | ATGATGGGCTGGYATTCT |
| PRRSV-R | ACACGGTCGCCCTAATTG |
| PRV-F | ACGGCACGGGCGTGATC |
| PRV-R | ACTCGCGGTCCTCGAGCA |
| VSV-F | ACGGCGTACTTCCAGATGG |
| VSV-R | CTCGGTTCAAGATCCAGGT |
| **Recombinant expression** | |
| pCAGGS-Flag-p72-F | ATGACGACGATAAGGAATTCATGGCATCAGGAGGAGCTTT |
| pCAGGS-Flag-p72-R | AAAAGATCTGCTAGCTCGAGTTAGGTACTGTAACGCAGCA |
| pCAGGS-HA-p72-F | TTCCAGATTACGCTGAATTCATGGCATCAGGAGGAGCTTT |
| pCAGGS-HA-p72-R | AAAAGATCTGCTAGCTCGAGTTAGGTACTGTAACGCAGCA |
| pCAGGS-HA-CD1d-F | ACGACGTCCCAGACTACGCTTTCCCCCTCCGCTGCCTCCA |
| pCAGGS-HA-CD1d-R | AAAAGATCTGCTAGCTCGAGTCACTGGATGTTTTGATAGA |
| pCAGGS-HA-CD1dΔC-F | ACGACGTCCCAGACTACGCTTTCCCCCTCCGCTGCCTCCA |
| pCAGGS-HA-CD1dΔC-R | AGATCTGCTAGCTCGAGTTAGACACGGCGCCTCCTAAACC |
| pCAGGS-Flag-CD1d-F | ATGACGACGATAAGGAATTCATGGATCTGCATTCCCTTCC |
| pCAGGS-Flag-CD1d-R | GAAAAAGATCTGCTAGCTCGAGTTACTGGATGTTTTGATACACCAAGACATGACTGTGGT |
| pCAGGS-Flag-EPS15-F | ATGACGACGATAAGGAATTCATGGCTGCGGCGGCCCAGCTTTCCCTGACACAGTTATCA |
| pCAGGS-Flag-EPS15-R | AAAAGATCTGCTAGCTCGAGTCATGCTTCTGAGATCTCAGATTTGCTGAGTGCAATAGC |
| pCAGGS-dsRed-EPS15-F | TCTCATCATTTTGGCAAAGAATTCATGGCTGCGGCGGCCCAGCTTTCC |
| pCAGGS-dsRed-EPS15-R | TGATGACGTTCTCGGAGGAGGCGAGCTCGAATTCTGCTTCTGAGATCTCAGA |
| **Overexpression of CD1d in non-permissive cells** | |
| pLVX-IRES-HA-CD1d-F | GGATCTATTTCCGGTGAATTCGCCACCATGGGGTACCTGCCGTTTGTGTT |
| pLVX-IRES-HA-CD1d-R1 | AGCGTAGTCTGGGACGTCGTATGGGTACATCTGGATGTTTTGATAGACAC |
| pLVX-IRES-HA-CD1d-R2 | GAGAGGGGCGGGATCCGCGGCCGCTCAAGCGTAGTCTGGGACGTC |
| **Knockout of CD1d** | |
| gRNA-CD1d-F | GCTGGCTGTGAGGTGCACCCGTTTTAGAGCTAGAAATAGCA |
| gRNA-CD1d-R | GGGTGCACCTCACAGCCAGCCGGTGTTTCGTCCTTTCCACA |
| pMJ-gRNA-MluI-F | GCCAGATATACGCGTACGCGTTGTACAAAAAAGCAGGCTTT |
| pMJ-gRNA-MluI-R | TCAATAATCAATGTCAACGCGTGGTGACACTATAGAATACT |

# 
